# Biogeography of the Southern Ocean: environmental factors driving mesoplankton distribution South of Africa

**DOI:** 10.7717/peerj.11411

**Published:** 2021-05-10

**Authors:** Alexander Vereshchaka, Eteri Musaeva, Anastasiia Lunina

**Affiliations:** Shirshov Institute of Oceanology, Russian Academy of Sciences, Moscow, Russia

**Keywords:** Zooplankton, Southern ocean, Distribution, Environmental factors

## Abstract

Spatial distribution of zooplankton communities depends on numerous factors, especially temperature and salinity conditions (hydrological factor), sampled depth, chlorophyll concentration, and diel cycle. We analyzed and compared the impact of these factors on mesoplankton abundance, biodiversity, quantitative structure based on proportion of taxa and qualitative structure based on presence/absence of taxa in the Southern Ocean. Samples (43 stations, three vertical strata sampled at each station, 163 taxa identified) were collected with a Juday net along the SR02 transect in December 2009. Mesoplankton abundance in discrete vertical layers ranged from 0.2 to 13,743.6 ind. m^−3^, i.e., five orders of magnitude, maximal and minimal values were recorded in the upper mixed and in the deepest layer, respectively. Within the combined 300-m layer, abundances ranged from 16.0 to 1,455.0 ind. m^−3^, i.e., two orders of magnitude suggesting that integral samples provide little information about actual variations of mesoplankton abundances. A set of analyses showed that depth was the major driver of mesoplankton distribution (abundance, biodiversity, quantitative structure), hydrological factors influenced two of them (quantitative and qualitative structure), chlorophyll concentration strongly affected only quantitative structure, and diel cycle had an insignificant effect on mesoplankton distribution. Using our current knowledge of the fine structure of the Antarctic Circumpolar Current, we compared effects of four hydrological fronts, i.e., boundaries between different water-masses with distinct environmental characteristics, and eight dynamic jets (narrow yet very intense currents) on mesoplankton distribution. Subtropical, Polar, and Subantarctic Fronts drove quantitative and qualitative structure of mesoplankton assemblages (decreasing in order of influence), while the Southern Boundary affected only qualitative structure. Effects of dynamic jets were insignificant. We suggest that mesoplankton composition is driven by hydrological parameters and further maintained through compartmentalization by fronts. Impact of local eddies and meanders on biodiversity, abundance, qualitative and quantitative structure of mesoplankton is comparable to that of hydrological fronts. Qualitative structure of mesoplankton assemblages mirrors hydrological structure of the Southern Ocean better than quantitative structure and may be recommended for biogeographic analyses of the Southern Ocean. Comparisons with previous reports from the same area retrieved no significant changes in mesoplankton distribution during the period 1992–2009.

## Introduction

Mesoplankton of the Southern Ocean is the key trophic link between phytoplankton microbial production and consumers such as fish, cephalopods, seabirds, and marine mammals (Atkinson, 1998a, 1998b; Pinkerton & Bradford-Grieve, 2014). Excretion at depth, egestion, and sinking corpses facilitate the transfer of organic matter to the ocean interior and energetically support benthic communities (Accornero & Gowing, 2003; Steinberg & Landry, 2017). By grazing on lower trophic levels and forming sinking particles, mesoplankton plays an important role in establishing export carbon regimes in the Southern Ocean (Halfter et al., 2020). Mesoplankton is thus a part of the Biological Carbon Pump, which is a critical component of climate regulation (Lumpkin & Speer, 2007; Mayewski et al., 2009).

Zooplankton distribution is influenced by numerous environmental factors but the impact of some factors is difficult to assess. Temperature, salinity, depth, and diel cycle are the only factors that have been widely studied in relation to plankton assemblages (Longhurst, 1976; Labat et al., 2009; Lebourges-Dhaussy et al., 2009; Constable et al., 2014; Lucas et al., 2014). There are recent evidences that other environmental factors, such as sea ice and climate indices, may also drive distribution of pteropods (Thibodeau et al., 2019) and macroplankton (Steinberg et al., 2015), at least near the Western Antarctic Peninsula. As the Southern Ocean is the most remote part of the World Ocean, the impact of environmental factors on plankton assemblages in this region has been studied less than in other oceanic areas. The Discovery Expedition of 1901–1904 (the British National Antarctic Expedition) laid the basis for our knowledge of the biology of the Southern Ocean and resulted in clarification of taxonomic composition, life cycles and distribution of principal plankton groups, such as copepods (Atkinson, 1991; Ommanney, 1936), chaetognaths (David, 1955, 1958), and various macroplankton taxa (Mackintosh, 1937). Later, the Discovery Expedition data were used for assessment of the plankton standing stock in the Southern Ocean by Foxton (1956), who found seasonal variations and a biomass increase near the Polar Front. Since the beginning of the 21st century, several detailed epipelagic studies in the Southern Ocean have shown the dependence of zooplankton characteristics on abiotic gradients associated with hydrological fronts (Pakhomov et al., 2000, 2006, Pakhomov & Froneman, 2004a, 2004b; Froneman, 2000; Ward et al., 2003; Jasmine et al., 2009). Further analysis of the Discovery data showed similar relationships for deeper mesopelagic layers (Ward et al., 2014). It is noteworthy that recent studies of plankton distribution in the Southern Ocean have been conducted using continuous plankton recorders (Labat et al., 2002, 2009; Takahashi et al., 2002), which provided robust statistics for gross zooplankton characteristics (abundance, biomass), but did not resolve structure of plankton assemblages at the species level. The use of plankton nets followed by the identification of animals is time-consuming, but it is still the only method yielding new detailed representative findings at the species level (Kulagin, 2010; Stupnikova & Vereshchaka, 2013; Stupnikova et al., 2018; Vedenin et al., 2019, 2020).

Historically, two fronts in the Southern Ocean have been defined as the boundary between two zones with distinct water-mass properties: the Subantarctic Front (SAF) and the Polar Front (PF) (Pakhomov et al., 2000, Pakhomov & Froneman, 2004a, 2004b; Froneman, 2000; Ward et al., 2003; Deacon, 1937; Nowlin & Clifford, 1982). The Antarctic Circumpolar Current (ACC) area also encompasses the Subtropical Front (STF) to the north and the Southern Boundary Front (SB) near Antarctica. In this paper we analyze impact of these four hydrological fronts taking into account that dynamics of the STF and the SB are distinct from the main ACC fronts (Chapman et al., 2020).

Now we know that, in addition to classic hydrological fronts, the ACC also includes a system of additional individual jets acting as dynamic fronts and corresponding to sea surface height (SSH) gradients (i.e., intense geostrophic velocities), not sea surface temperature (SST) gradients. Some jets coincide with hydrological fronts, some are lacking sharp gradients of temperature and salinity and thus not synonymous with classic hydrological fronts (Burkov, 1994; Orsi, Whitworth & Nowlin, 1995; Tarakanov & Gritsenko, 2014). Here we note that there is no agreement in the status of the Southern ACC Front (SACCF), which is either recognized as a hydrological front (e.g., Chapman et al., 2020) or considered as strictly a dynamic jet (Tarakanov & Gritsenko, 2014).

In this paper we tested and hierarchized major environmental factors (hydrological proprieties, depth, diel cycle, chlorophyll concentration) that are responsible for differences between plankton assemblages and further compared the effects individual hydrological fronts and dynamic jets have on the plankton assemblages. With a deeper insight into biogeography of the Southern Ocean, we will better understand important ecosystem services, such as climate regulation and nutrient recycling in such a significant region as the Subantarctic and Polar frontal zones of the ACC area (Halfter et al., 2020).

The survey used in this study is based on net samples and aimed to create a detailed analysis of the relationships between environmental factors and the abundance/biodiversity of 129 plankton assemblages. In addition to traditionally studied environmental factors, such as temperature, salinity, hydrological zones (these parameters are highly correlated in the Southern Ocean), depth, and diel cycle, we also tested chlorophyll concentration. Surface chlorophyll-a concentration (*Chl*) recorded by satellite was used as a proxy of biological productivity and can explain characteristics of plankton communities (Vereshchaka et al., 2016, 2017; Vereshchaka, Lunina & Sutton, 2019). In this paper, we first compare the impact the environmental factors listed above have on mesoplankton structure in the Southern Ocean.

Another novelty of this paper is the study of the comparative effect of hydrological fronts (boundaries between different water-masses with distinct environmental characteristics) and dynamic jets (narrow yet very intense currents) on the plankton assemblages in the Southern Ocean.

Finally, our survey was made nearly “two decades later” the survey of the same area during the austral summer 1992/1993 (Pakhomov et al., 2000). This previous survey was focused on the same plankton group and the same layer 0–300 m and comparison of both datasets may reveal possible changes (or their absence) in mesoplankton associations in the Southern Ocean.

## Methods

### Hydrological setting

**Figure 1:**
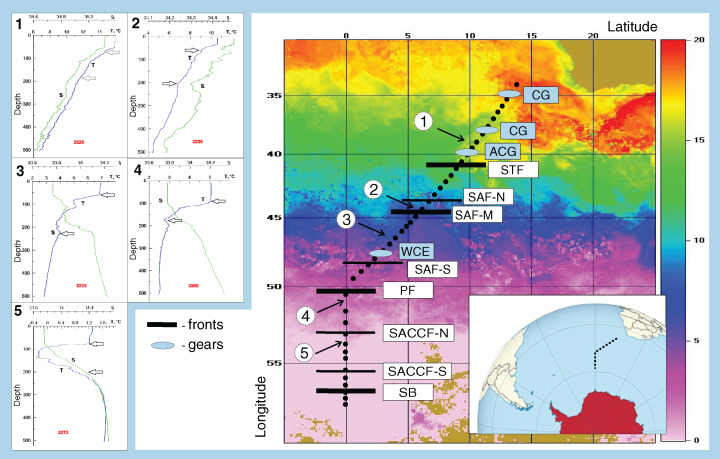
Study area and stations on temperature field (averaged from 30 November to 31 December 2009). Hydrological jet coding: Subtropical Front (STF); Subantarctic Front (SAF), three branches (northern SAF-N, middle SAF-M and southern SAF-S); Polar Front (PF), merged branches; Southern ACC Front (SACCF), two branches (northern SACCF-N and southern SACCF-S); Southern Boundary (SB). Gyre coding: cyclonic gyres (CG), anticyclonic gyres (ACD), warm core eddy (WCE). Characteristic T-S profiles provided for the hydrological zones bounded by jets: arrows indicate vertical boundaries of net hauls and station numbers are in red. Position of fronts and gyres: interpretation after Tarakanov & Gritsenko (2014).

The ACC was shown to consist of nine (Sokolov & Rintoul, 2009) or even more (Tarakanov & Gritsenko, 2014) principal jets or frontal filaments. Sokolov & Rintoul (2009) identified the following jets listed from north to south: three branches of the SAF (northern SAF-N, middle SAF-M and southern SAF-S), three branches of the PF (northern PF-N, middle PF-M and southern PF-S), two branches of the SACCF (northern SACCF-N and southern SACCF-S), and a single jet of the SB. Hereafter, we used “fronts” for boundaries between waters masses with sharp hydrological gradients and “jets” for narrow, dynamic fronts. We surveyed the SR02 hydrophysical transect (submeridional, from the South Africa to the Weddell Sea–Fig. 1), where most jets and fronts were well-recognizable, owing to the ACC width and relatively low influence of the bottom relief (Tarakanov & Gritsenko, 2014). Since hydrological boundaries are known to vary along longitude and the individual branches can merge and diverge (Sokolov & Rintoul, 2009), our sampling was synchronized with a detailed hydrographic survey. Hydrological in situ data (temperature, salinity, density) were further combined with satellite information and analyzed in detail by Tarakanov & Gritsenko (2014). All ACC structures sensu Sokolov & Rintoul (2009) were present, except individual filaments of the PF, which merged and formed a single jet. The zone of the STF was not continuous and consisted of cyclonic and anticyclonic circulations of different genesis, sometimes incorporating Subantarctic waters (Sokolov & Rintoul, 2009; Tarakanov & Gritsenko, 2014). Using direct measurements (SeaBird 911+ and SBE-21 CTD profilers, a ship-borne Doppler acoustic profiler of the currents SADCP, TRDI Ocean Surveyor, 38 kHz) and the ADT satellite data (the DT-Global-MADT-Upd product from the French agency Collecte Localisation Satellites available at the site http://aviso.oceanobs.com) along the transect, Tarakanov & Gritsenko (2014) identified 11 jets, eight of which were identical to dynamic jets sensu Sokolov & Rintoul (2009): a single STF, three branches of the SAF, a single PF, two branches of the SACCF, and the SB. We further analyzed the impact of these eight dynamic jets. Tarakanov & Gritsenko (2014) also recorded four hydrological fronts associated with the STF, the SAF-M, the PF, and the SB jets (no gradients in the SACCF jets), which we will further consider as fronts. In addition to fronts and jets, we crossed two cyclonic and one anticyclonic gyres north of the STF (identified and discussed by Tarakanov & Gritsenko, 2014) and a single warm core eddy between SAF-M and SAF-S not considered in Tarakanov & Gritsenko (2014) but clearly visible in our T-S dataset.

### Sample collection and analysis

Samples were taken during the 30^th^ Cruise of the R/V ‘Akademik Ioffe’ along the SR02 transect (WOCE nomenclature, Fig. 1) between 34.44° S and 56.90° S, from December 5–22, 2009, with intervals of 10–15 h (Fig. 1, Appendix 1). At each station, CTD casts were performed using a SeaBird 911+ profiler prior to biological sampling (see details in Tarakanov & Gritsenko, 2014). A total of 43 stations were sampled with a Juday net (0.1 m^2^ mouth area, 35 cm diameter, 180 µm mesh size), towed at ~1 m s^−1^.

We sampled mesoplankton in the active, upper 300 m of the water column (i.e., the whole epipelagic zone and the upper mesopelagic). In order to make hauls vertical, nets were weighted with a 60-kg sinker; the angle of inclination was always ≤ 20° and the spun cable (S_c_) was calculated for each haul as: S_c_ = D_t_ cos(α)^−1^, where D_t_ is target depth and α is angle of inclination. In order to assess the depth factor, we sampled three strata separated by vertical gradients of temperature and salinity at each site. We defined that the Juday net had a filtration coefficient equal to 1 and estimated volume of water filtered (V) as: V (m^3^) = 0.1 * L, where L (m) is the difference in the spun cable between the beginning and end point of sampling and 0.1 (m^2^) is the mouth area. The net was equipped with the closing device and an operator, which sometimes resulted in insignificant overlapping (<10% of depth ranges) of sampled layers.

The uppermost mixed layer was well-defined and bounded from below by seasonal halo- and thermoclines; two deeper layers were separated from each other by maximal gradients of temperature and salinity between the thermocline and 300 m depth. Actual sampling depths ranged along the transect; the upper and intermediate layers usually represented the epipelagic zone, while the deep layer occurred mainly in the upper mesopelagic (Fig. 1, Appendix 1). On average, in the upper, intermediate, and deep layers we filtered 6.20 ± 1.93 m^3^, 12. 31 ± 2.76 m^3^, and 11.76 ± 2.54 m^3^, respectively (Mean ± SD).

Samples were preserved in 4% seawater/buffered formaldehyde solution (250–500 ml each sample) and identified to the lowest possible taxonomical level using a stereomicroscope. In order to avoid bias linked to unrepresentative sampling of larger organisms (jellyfishes, salps, and euphausiids over 10 mm total length), we excluded these groups from analyses. Most taxa were counted in the whole sample; abundant taxa, such as *Oithona, Oncaea*, nauplii, and small copepodites were counted in the aliquots, which were sub-sampled with Stempel pipettes. In the aliquots (1/10–1/2 of the total sample), a minimum 100 individuals of each taxon were counted.

### Mesoplankton abundance and diversity indices used

We analyzed quantitative (QNT) and qualitative (QUAL) structure of plankton assemblages using quantitative and qualitative Bray–Curtis indices, respectively. The quantitative Bray–Curtis was based on an untransformed dataset and described taxa proportion in the assemblages and quantitative mesoplankton structure. The qualitative index was based on a presence-absence transformation of the dataset and sensitive to rare species and better mirrored all recorded taxa. We used qualitative indices because tests based on original abundance values are often over-dominated by a small number of highly abundant species and therefore fail to reflect overall community composition (Clarke & Warwick, 2001). This argument may be particularly true for our dataset: the survey was made in December, when most seasonal migrants ascended into the upper layer and a complete set of species was present in plankton assemblages (Hardy & Gunther, 1935; Mackintosh, 1934; Voronina, 1984). However, the transect crossed hydrological zones with different biological seasons, and abundances of some taxa could be underestimated in higher latitudes, where populations still partially occurred below 300 m depth. Indeed, recent use of qualitative approach in the analyses of plankton communities of the Drake Passage resulted in a more comprehensible outcome than the use of quantitative indices (Vedenin et al., 2019).

Biodiversity was estimated using the taxa number, and Shannon (*H*) and Dominance (*D*) indices. *H* (entropy) varies from 0 for communities with only a single taxon to high values for communities with many taxa, each with few individuals. *D* ranges from 0 (all taxa are equally represented) to 1 (one taxon dominates the community completely). Both indices were calculated as

$$H^{}=-\overset{R}{\underset{i=1}{\sum }}p_{i}ln\;p_{i}$$

$$D=\overset{R}{\underset{i=1}{\sum }}{\left(p_{i}\right)}^{2}$$

where *p*_*i*_ is the proportion of individuals belonging to the *i*-th species in the dataset with *R* taxa.

### Determining the impact of environmental factors on mesoplankton abundance and biodiversity

Canonical Correspondence Analyses (CCAs: Ter Braak, 1986; Legendre & Legendre, 1998) were conducted in order to compare the impact of major environmental factors on plankton communities. We scored four environmental factors and divided each factor by ordinals:

- Hydrological zones/temperature/salinity: For each station, we collected information about temperature and salinity at the surface and at a depth of 300 m (deepest sampled depth). In addition, we calculated average temperature and salinity for each sampled vertical layers on the basis of temperature profiles (Appendix 1). In order to assess and compare effects of hydrological fronts and jets, we completed two analyses: we first divided the transect into five zones bounded by four hydrological fronts (Analysis 1) and then into nine zones bounded by eight jets (Analysis 2–Table 1). If a station was located exactly on a jet or on a front boundary, it was referred to both neighboring zones. In our dataset, temperature and salinity values and hydrological zones were highly correlated. In order to avoid collinearity in the CCAs, we chose hydrological zones and considered them as ordinals: 1–5 in Analysis 1 and 1–9 in Analysis 2. Hereafter, the terms “hydrological zone” will refer to a region bounded by two fronts or jets, and “hydrological factor” will refer to temperature and salinity, keeping in mind that either of these variables (or likely both) contribute to the effect of this factor.
- Depth: Sampled strata were coded as ordinals: 1 (upper mixed layer), 2 (intermediate layer), and 3 (deep layer).
- Diel cycle: Conditions were expressed by three ordinals: 1 (night), 2 (dusk), and 3 (day). Dusk was defined as the time period between one hour before and one hour after sunrise or sunset, local time.
- Chlorophyll concentration (*Chl*): The data were retrieved from satellite imaging and were used as a proxy of biological productivity. The values were obtained from Aqua MODIS scanner (level 3, 4-km resolution, https://oceancolor.gsfc.nasa.gov/) and averaged over the month of the survey (December 2009) and over a 1° (latitude) × 5° (longitude) rectangle with the sampling site in the center. *Chl* values were further divided into six ranges and scored as ordinals: 1 (0.20–0.25 mg m^−3^), 2 (0.25–0.30 mg m^−3^), 3 (0.30–0.35 mg m^−3^), 4 (0.35–0.40 mg m^−3^), 5 (0.40–0.45 mg m^−3^), and 6 (0.45–0.50 mg m^−3^).

**Table 1:** List of dominant taxa (>0.1% contribution to the total abundances).

Taxa	Contribution, %
1. *Oithona similis*	49.20
2. Calanoida copepodites indet.	11.86
3. Euphausiidae nauplii	4.91
4. *Ctenocalanus citer*	4.89
5. Foraminifera	4.62
6. Ova (copepoda)	3.06
7. *Microcalanus pusillus*	2.82
8. Appendicularia	2.70
9. Radiolaria	2.04
10. *Clausocalanus brevipes*	1.36
11. *Triconia antarctica* ♂	1.29
12. *Clausocalanus pergens*	1.10
13. *Paracalanus parvus parvus*	0.81
14. *Oncaea* sp.	0.67
15. *Pleuromamma gracilis*	0.65
16. Ostracoda	0.56
17. *Metridia lucens lucens*	0.49
18. Chaetognatha	0.45
19. *Acartia longiremis*	0.43
20. Copepoda nauplii	0.43
21. *Calanus simillimus*	0.42
22. *Calocalanus contractus*	0.39
23. Pteropoda	0.39
24. *Scolecithricella minor*	0.35
25. *Oithona plumifera*	0.34
26. *Ctenocalanus vanus*	0.31
27. *Calocalanus styliremis*	0.30
28. *Calanus propinquus*	0.29
29. *Euchaeta marina*	0.25
30. *Triconia antarctica* ♀	0.23
31. *Onychocorycaeus giesbrechti*	0.19
32. Euphausiacea calyptopis	0.19
33. Salpidae	0.18
34. *Clausocalanus laticeps*	0.15
35. Euphausiacea furcilia	0.14
36. *Rhincalanus gigas*	0.11

All four factors were not correlated (pairwise correlations were checked).

We used pairwise two-way analyses of similarities (ANOSIM) to determine the difference between samples grouped by hydrological zones, depth, diel cycle, and *Chl* ranges. We also used an ANOSIM test for a pairwise comparison between stations bounded by hydrological fronts (Analysis 1) and dynamic jets (Analysis 2).

We used traditional plots of abundance and diversity in the depth-latitude coordinates to visualize the impact of depth and hydrological zones. The plots were made using the SURFER package (v. 11.0.642, grid method: kriging, smoothing: low) in order to show trends in distribution.

For a more in depth analysis of the impact of hydrological boundaries, we assessed the discontinuities of plankton communities along the transect. We used a number of unique taxa (NUT), which were present at a given station and absent at both neighboring stations. NUT values were smoothed using the Gaussian, “nine points” algorithm.

The Bray–Curtis matrices were also analyzed using hierarchical clustering, the Unweighted Pair-Group Method (UPGMA). Results were verified by Similarity Profile Analysis (SIMPROF) to distinguish different groups with significant differences in variables (Clarke & Warwick, 2001).

Statistics were performed using Primer v6, Past 3, Surfer 15 and Microsoft Excel 2010 software (Clarke & Warwick, 2001; Hammer, Harper & Ryan, 2008). We accepted a significance level of *p* = 0.05.

## Results

### Composition of zooplankton assemblages and distribution of dominant taxa

We identified 163 taxa, predominately copepods (dominant taxa listed in Table 1, full list in Appendix 2). *Oithona similis* composed nearly half of the mesoplankton abundance and was followed by four other taxa, each contributing >4% to the total abundance: unidentified young calanoid copepodites, euphausiid nauplii, *Ctenocalanus citer*, and Foraminifera. These five taxa accounted for over 75.49% of the total mesoplankton abundance.

**Figure 2:**
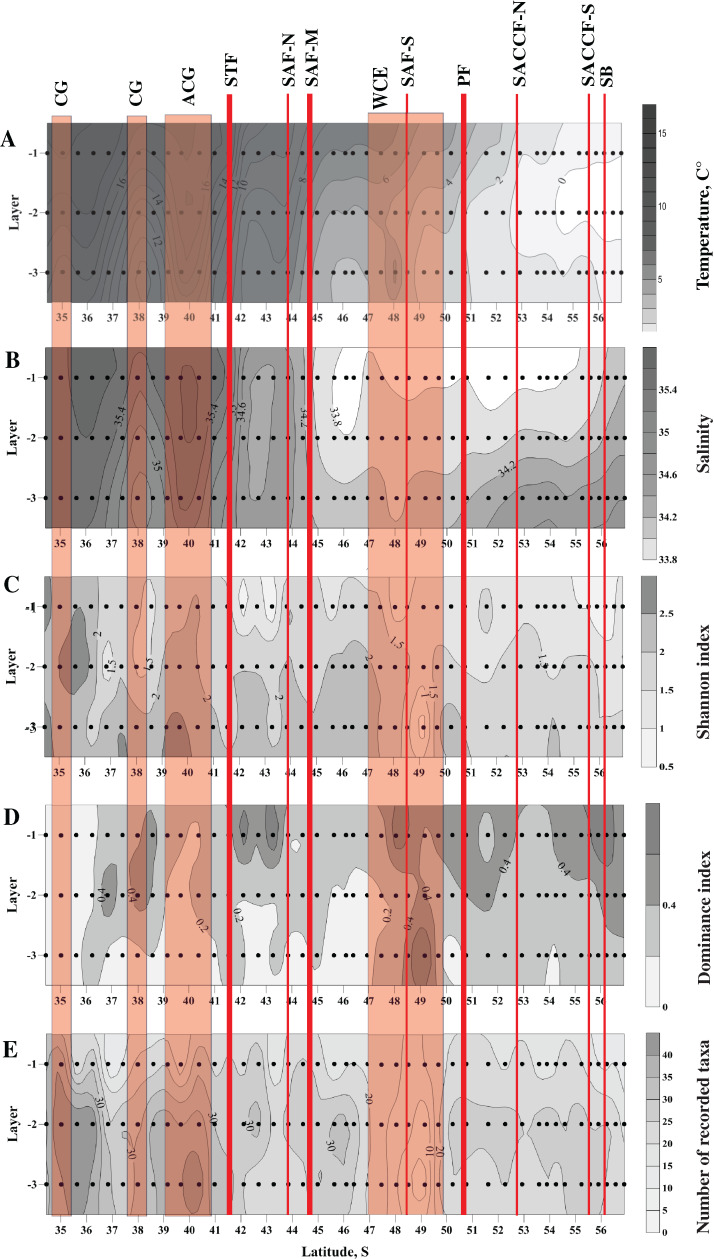
Distribution of temperature (C°) and salinity averaged for sampled layers (A and B, respectively), Shannon and Dominance biodiversity indices (C and D, respectively), and number of recorded taxa (E); scales presented on the right of the plots. Dots indicate the position of the samples. Vertical layer coding is as follows: upper mixed layer (1), intermediate layer (2), deep sampled layer (3). Hydrological jet coding (vertical red lines): Subtropical Front (STF); Subantarctic Front (SAF), three branches (northern SAF-N, middle SAF-M and southern SAF-S); Polar Front (PF), merged branches; Southern ACC Front (SACCF), two branches (northern SACCF-N and southern SACCF-S); Southern Boundary (SB). Gyre coding (vertical opaque red strips): cyclonic gyre (CG) and anticyclonic gyre (ACG). Position of fronts and gyres: interpretation after Tarakanov & Gritsenko (2014). SURFER 11.0.642, grid method: kriging, smoothing: low.

Distribution of all three biodiversity indices suggested the highest biodiversity north of 37° S (Figs. 2C–2E), i.e., in the core waters of the Agulhas Current (Figs. 2A, 2B). Within the ACC, biodiversity indices did not show visible horizontal trends along the transect, except a plankton-depleted gap between 47° and 50° S (Figs. 2C–2E). Highest biodiversity was recorded in the deep layer (maximal *H* and minimal *D* values), i.e., evenness of planktonic assemblages grew with depth (Figs. 2C–2E).

**Figure 3:**
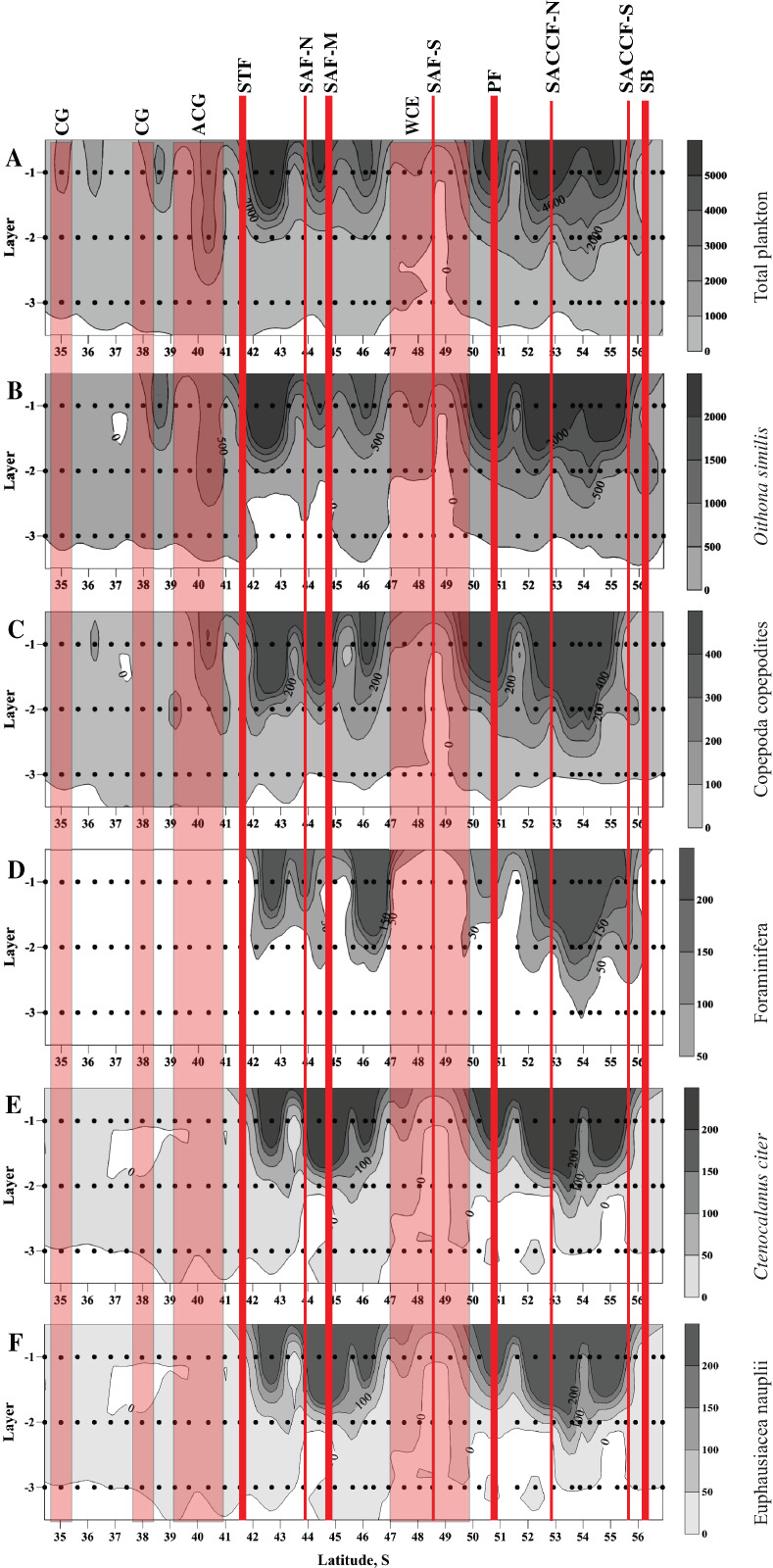
Distribution of total plankton abundance (A), abundances of *Oithona similis* (B), Copepoda copepodites (C), Foraminifera (D), *Ctenocalanus citer* (E), and Euphausiacea nauplii (F). Scales presented on the right of the plots (abundance: ind. m^−3^). Dots indicate position of samples. Vertical layer coding: upper mixed layer (1), intermediate layer (2), deep layer (3). Hydrological jet coding (vertical red lines): Subtropical Front (STF); Subantarctic Front (SAF), three branches (northern SAF-N, middle SAF-M and southern SAF-S); Polar Front (PF), merged branches; Southern ACC Front (SACCF), two branches (northern SACCF-N and southern SACCF-S); Southern Boundary (SB). Gyre coding (vertical opaque red strips, interpretation after Tarakanov & Gritsenko (2014)): cyclonic gyre (CG) and anticyclonic gyre (ACG). Position of fronts and gyres: interpretation after Tarakanov & Gritsenko (2014). SURFER 11.0.642, grid method: kriging, smoothing: low.

High abundances of mesoplankton, including all five dominant taxa, were generally observed between the STF and the SB, with a notable gap between 47° and 50° S (Figs. 3A–3F). Increased abundances of the total mesoplankton, *O. similis*, and calanoid copepodites were locally observed north of the STF and were associated with the subtropical anticyclonic gyre (Figs. 3A–3C). In contrast to biodiversity indices, highest abundance of dominant taxa was recorded mostly in the upper mixed layer.

No notable changes in biodiversity indices, abundance of dominant taxa and total mesoplankton abundance were visibly associated with individual hydrological fronts or jets.

### Comparative impact of major environmental factors on mesoplankton abundance and biodiversity

**Figure 4:**
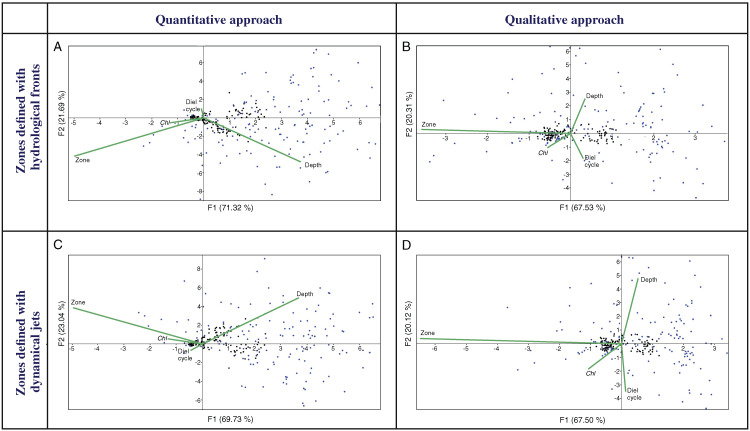
CCAs with stations bounded by four hydrological fronts and eight dynamic jets, under quantitative data based on proportion of taxa (A and C), stations bounded by four hydrological fronts and qualitative data based on presence/absence of taxa (B and D), stations bound. Two first axes (F1 and F2) with respective variance are represented. Environmental variables: hydrological zone (Zone), depth layer (Depth), surface chlorophyll averaged over the month of the survey (December) and over a 1° (latitude) × 5° (longitude) rectangle with the sampling site in the centre (*Chl*), and diel cycle conditions (Diel cycle). Black circles indicate position of samples, and blue circles indicate position of taxa.

Multivariate CCAs using a quantitative approach (QNT-structure of mesoplankton) showed similar results for Analysis 1 (stations bounded by hydrological fronts, Fig. 4A) and Analysis 2 (stations bounded by jets, Fig. 4C). Two first major factors (thereafter named F1, F2) explained together almost 92% of the variance of the dataset and they were subequally linked to hydrological zone and depth and to a lesser extent with *Chl*. Diel cycle factor was insignificant.

CCAs using a qualitative approach (QUAL-structure of mesoplankton) also showed similar results for Analysis 1 and Analysis 2 (Figs. 4B and 4D), but these results were significantly different from those obtained with the quantitative approach. Factor F1 explained ~67% of variance and was strongly linked only to hydrological zone. The three other factors were subequal and less significant.

Pairwise two-way ANOSIM tests confirmed results of CCAs and revealed constant statistically significant differences between assemblages bounded by hydrological zone, depth, and *Chl* (Table 2). Both quantitative and qualitative approaches resulted in a robust difference provided by these three factors in all combinations. Diel cycle did not provide a statistically significant separation in any of the pairwise combinations, which is in agreement with the results of CCAs. In both two-way ANOSIM tests and CCAs the results are the same for hydrological zones delimited by fronts or jets. The specific difference between fronts and jets in shaping the distribution of zooplankton assemblages is further explored in the following section.

**Table 2:** Results of the pairwise tests (two-way ANOSIM) used to identify significant differences between plankton assemblages sampled in different depth layers (Depth), hydrological zones (Zone) divided by four fronts or eight jets, during different diel cycles (Diel), and in different productive zones (*Chl*). Combinations Zone-*Chl* are not included due to insufficient group level combinations. Significant *p*-values are in bold.

Pairwise test	Factors	Bray-Curtis quantitative	Bray-Curtis qualitative
Depth-Zone (four fronts)	Depth, p	**0.0001**	**0.0001**
	Zone, p	**0.0001**	**0.0001**
Depth-Zone (eight jets)	Depth, p	**0.0001**	**0.0001**
	Zone, p	**0.0001**	**0.0001**
Diel-Zone (four fronts)	Diel, p	0.8306	0.5855
	Zone, p	**0.0001**	**0.0001**
Diel-Zone (eight jets)	Diel, p	0.8722	0.2573
	Zone, p	**0.0050**	**0.0001**
Diel-Depth (four fronts)	Diel, p	0.6509	0.6359
	Depth, p	**0.0001**	**0.0072**
Diel-Depth (eight jets)	Diel, p	0.8013	0.4202
	Depth, p	**0.0001**	**0.0015**
Chl-Diel (four fronts)	Chl, p	**0.0041**	**0.0002**
	Diel, p	0.148	0.6889
Chl-Diel (eight jets)	Chl, p	**0.0040**	**0.0002**
	Diel, p	0.1249	0.5493
Chl-Depth (four fronts)	Chl, p	**0.0031**	**0.0001**
	Depth, p	**0.0001**	**0.0002**
Chl-Depth (eight jets)	Chl, p	**0.0031**	**0.0009**
	Depth, p	**0.0001**	**0.0002**

### Comparative impact of hydrological fronts and dynamic jets on mesoplankton abundance and biodiversity

**Figure 5:**
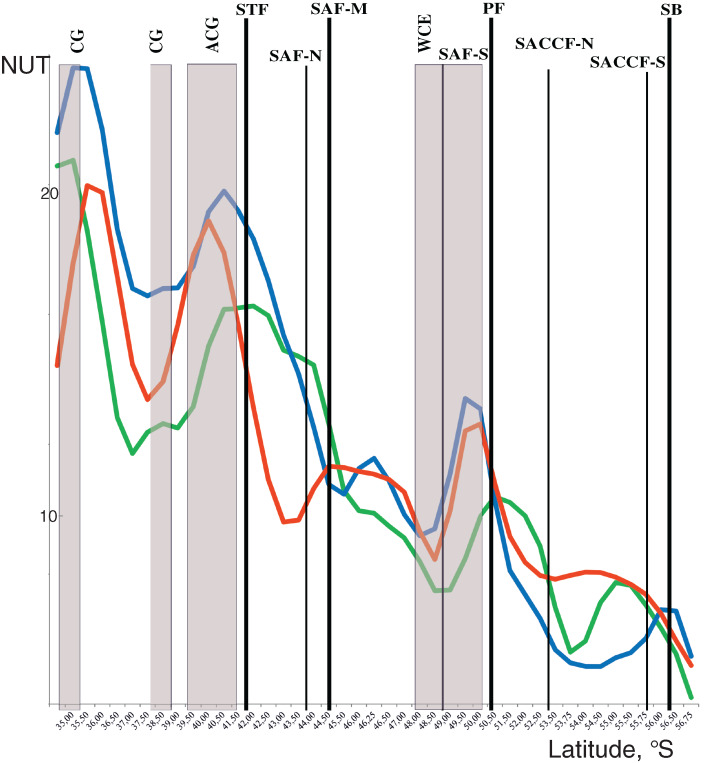
Discontinuity of plankton assemblages along transect: number of unique taxa respective neighboring stations (NUT). Gaussian smoother for nine points used for upper layer (green line), intermediate layer (blue line), and deep layer (red line). Hydrological coding (vertical black lines): Subtropical Front (STF); Subantarctic Front (SAF), three branches (northern SAF-N, middle SAF-M and southern SAF-S); Polar Front (PF), merged branches; Southern ACC Front (SACCF), two branches (northern SACCF-N and southern SACCF-S); Southern Boundary (SB), cyclonic gyre (CG), anticyclonic gyre (ACG) and warm core eddy (WCE). Hydrological fronts are indicated by thicker lines.

Discontinuities of assemblages assessed through the NUT (number of the unique taxa) index showed a general southward decrease along the transect from maximal values at two northernmost stations and minimal values at the southernmost station. Two remarkable maxima were, however, observed along the transect with different position in the upper mixed layer and underlying layers. In the upper layer, NUT maxima were associated with two hydrological fronts (the STF and the PF: green line in Fig. 5), while in deeper layers NUT maxima were recorded northernmore: within an anticyclonic gyre of the Agulhas Current near 40° S and the warm core eddy at ~40° S (blue and red lines in Fig. 5). There was no visible association of NUT peaks with the dynamic jets.

The ANOSIM tests (Table 3) confirmed the strong separating effect of the three hydrological fronts (STF, SAF-M, PF) and an insignificant effect of the dynamic jets. When we divided plankton assemblages into five groups bounded by hydrological fronts and used the qualitative approach (study of QUAL-stricture), all groups were significantly different from each other, except the fifth group bounded by the SB (ANOSIM tests in Table 3 and MDS scaling in Appendix 3). This effect was observed for each individual layer and for the whole 300-m layer with the exception of a boundary between the second and third groups in the deep layer. The quantitative approach (study of QNT-structure-Appendix 4) resulted in fewer statistically significant boundaries: the STF (all layers), the PF (intermediate and deep layers), and the SB (upper layer).

**Table 3:** Results of the ANOSIM tests to identify differences between plankton assemblages bounded by hydrological fronts (Bray-Curtis qualitative index used). Tested layers: upper mixed (Layer 1), intermediate (Layer 2), deep (Layer 3), and the whole 0-300 m layer (Layer 0). Zones: north of STF (1), between STF and SAF-M (2), between SAF-M and PF (3), between PF and SB (4), and south of SB (5). Statistically significant boundaries between neighboring zones are in bold.

**Layer 1**				
	2	3	4	5
1	**0.0002**	**0.0001**	**0.0001**	**0.0015**
2		**0.0006**	**0.0004**	**0.009**
3			**0.0002**	**0.0035**
4				0.0674
**Layer 2**				
	2	3	4	5
1	**0.0002**	**0.0001**	**0.0001**	**0.0022**
2		**0.0363**	**0.0001**	**0.009**
3			**0.0001**	0.2921
4				**0.0135**
**Layer 3**				
	2	3	4	5
1	**0.0002**	**0.0001**	**0.0001**	**0.0017**
2		0.2198	**0.0001**	**0.0082**
3			**0.0002**	0.2998
4				0.0691
**Layer 0**				
	2	3	4	5
1	**0.0001**	**0.0001**	**0.0001**	**0.0019**
2		**0.0018**	**0.0001**	**0.0068**
3			**0.0001**	**0.0441**
4				0.1205

When we divided plankton assemblages into nine groups bounded by jets and used the qualitative approach, the boundary effect was weaker and only a few combinations passed tests (ANOSIM tests in Table 4 and MDS scaling in Appendix 5): the STF in all layers, and the PF, the SACCF-N, and the SACCF-S in some layers. The SAF-N, the SAF-M, the SAF-S, and the SB jets did not provide statistically significant boundaries. Quantitative results (Appendix 6) indicated a significant difference in the boundary effect only twice: the STF (the deep layer) and the SACCF-S (upper layer).

**Table 4:** Results of the ANOSIM tests to identify differences between plankton assemblages bounded by jets (Bray-Curtis qualitative index used). Tested layers: upper mixed (Layer 1), intermediate (Layer 2), deep (Layer 3), and the whole 0-300 m layer (Layer 0). Zones: north of STF (1), between STF and SAF-N (2), between SAF-N and SAF-M (3), between SAF-M and SAF-S (4), between SAF-S and PF (5), between PF and SACCF-N (6), between SACCF-N and SACCF-S (7), between SACCF-S and SB (8), and south of SB (9). Statistically significant boundaries between neighboring zones are in bold.

**Layer 1**								
	2	3	4	5	6	7	8	9
1	**0.0017**	**0.0093**	**0.0001**	**0.0002**	**0.0004**	**0.0001**	**0.0014**	**0.0026**
2		0.8931	**0.0009**	**0.0089**	**0.0077**	**0.0013**	**0.0185**	**0.0197**
3			0.2753	0.068	**0.0345**	**0.0124**	0.0996	0.1057
4				0.3961	**0.0089**	**0.0016**	**0.008**	**0.0055**
5					**0.0363**	**0.0024**	**0.0335**	**0.0196**
6						0.1725	0.1877	0.0505
7							**0.0363**	**0.012**
8								0.3884
**Layer 2**								
	2	3	4	5	6	7	8	9
1	**0.0006**	**0.0026**	**0.0001**	**0.0001**	**0.0002**	**0.0001**	**0.0014**	**0.002**
2		0.8194	**0.0328**	**0.0183**	**0.0074**	**0.0014**	**0.0348**	**0.0163**
3			0.4522	0.1986	**0.0194**	**0.0117**	0.1006	0.1017
4				0.3213	0.212	0.0557	0.7609	0.1329
5					**0.0388**	**0.0025**	0.2827	0.1796
6						**0.0077**	**0.0182**	**0.0158**
7							0.0844	**0.0129**
8								0.499
**Layer 3**								
	2	3	4	5	6	7	8	9
1	**0.0004**	**0.0026**	**0.0001**	**0.0003**	**0.0001**	**0.0001**	**0.0017**	**0.0015**
2		0.3006	0.1063	**0.017**	**0.0085**	**0.0021**	**0.0172**	**0.0164**
3			0.773	0.5125	**0.0186**	**0.0105**	0.1022	0.1034
4				0.1771	0.2611	**0.0176**	0.2493	0.1615
5					0.1573	**0.0042**	0.2679	0.3388
6						0.1515	**0.0183**	**0.0178**
7							**0.047**	**0.0252**
8								0.3062
**Layer 0**								
	2	3	4	5	6	7	8	9
1	**0.0003**	**0.0017**	**0.0001**	**0.0002**	**0.0001**	**0.0001**	**0.0025**	**0.0016**
2		0.4412	**0.0019**	**0.0088**	**0.0069**	**0.0018**	**0.035**	**0.0173**
3			0.0743	0.1217	**0.0196**	**0.0102**	0.099	**0.1001**
4				0.0511	**0.003**	**0.0007**	**0.013**	**0.0128**
5					0.0853	**0.0023**	0.1218	0.1457
6						**0.0487**	**0.019**	**0.0183**
7							**0.0228**	**0.0119**
8								0.3976

**Figure 6:**
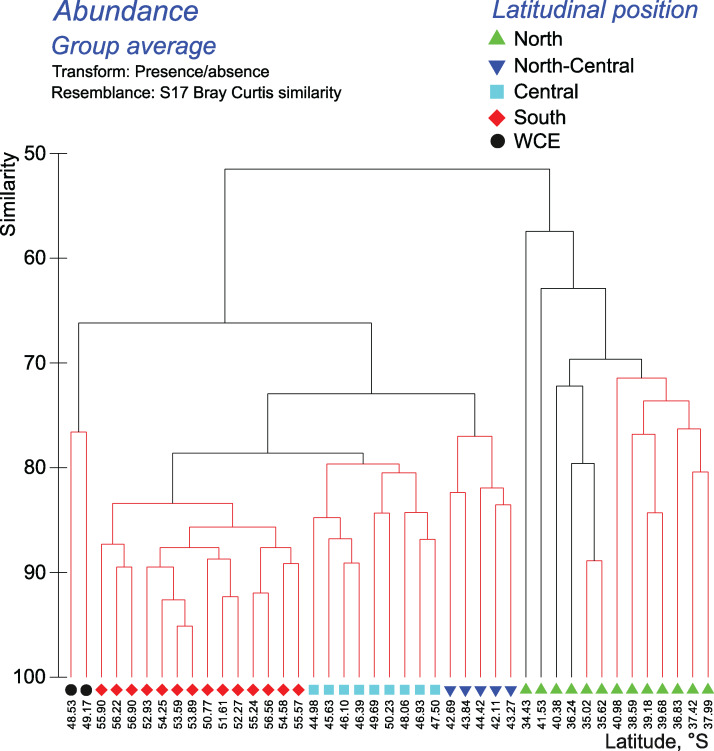
Results of the cluster analysis (Bray–Curtis qualitative index) of samples collected within the upper 300-m layers. Colored lines and nodes indicate robust (black, *p* < 0.05) and statistically insignificant (red, *p* > 0.05) clusters. Colored figures on bottom indicate robust clusters. Black dots indicate samples not grouped with other clusters.

**Figure 7:**
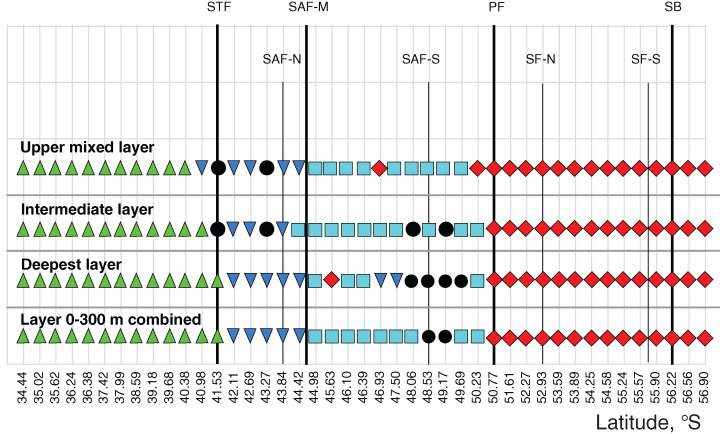
Distribution of the retrieved clusters (Bray-Curtis qualitative index, Fig. 6) along transect. Hydrological jet coding (vertical black lines): Subtropical Front (STF); Subantarctic Front (SAF), three branches (northern SAF-N, middle SAF-M and southern SAF-S); Polar Front (PF), merged branches; Southern ACC Front (SACCF), two branches (northern SACCF-N and southern SACCF-S); and Southern Boundary (SB). Hydrological fronts are shown by thicker lines. Colors indicate groups diverging at certain similarity levels. Black dots indicate samples not grouped with other clusters.

Cluster analyses confirmed the results of the discontinuity approach and ANOSIM tests. The qualitative approach retrieved a strong separating effect of all hydrological fronts on QUAL-structure of assemblages, except the SB (visible in all layers and in the whole 0–300 m layer–Figs. 6–7, Appendices 7–9). In the whole 0–300 m layer, four statistically significant clusters fitted four distinct assemblages between the fronts, while assemblages in the individual layers between the fronts were in some cases represented by one to three subclusters. The deepest node in cladograms always corresponded to the STF, suggesting the greatest separating effect by this front. It is notable that two 0–300 m samples (black dots) from the plankton gap in the warm core eddy were not grouped with other samples.

**Figure 8:**
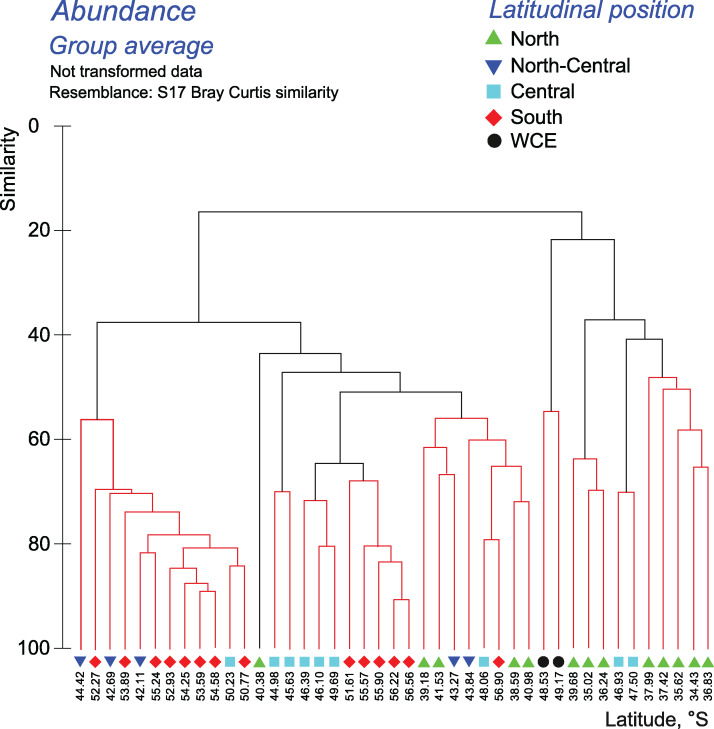
Results of the cluster analysis (Bray–Curtis quantitative index) of samples collected within the upper 300-m layers. Colored lines and nodes indicate robust (black, *p* < 0.05) and statistically insignificant (red, *p* > 0.05) clusters. Colored figures as in Fig. 6.

The quantitative approach showed that the effect of all hydrological fronts on QNT-structure of assemblages is present but obscure (Fig. 8, Appendices 10–11). For example, the left robust cluster at similarity level ~38 (Fig. 8) includes mainly South stations (south of the PF) interrupted by three North-Central stations (between the STF and the SAF) and a single Central station (between the SAF and the PF–Fig. 8). The right robust clade at similarity level ~38 (Fig. 8) encompasses mostly North stations (north of the STF) along with two Central stations.

Tables 5 and 6 show taxa, which contributed in differences between mesoplankton communities within the 0–300 m layer bounded by hydrological fronts. Differences in QUAL-structure are supported mainly by copepods (99 taxa) and, to a lesser extent, by amphipods (7 taxa). Differences in QNT-structure of assemblages are mainly explained by 11 copepod taxa (a total of ~70 %, with a leading role of *Oithona similis*), and unidentified Euphausiacea, Appendicularia, Foraminifera, and Radiolaria (2–4% each group).

**Table 5:** List of taxa contributing to differences between mesoplankton assemblages bounded by four hydrological fronts, qualitative approach based on presence/absence of taxa (retrieved in Figs. 6–7). 1/0–present/absent within assemblages.

Species	Presence/Absence			
	North	North-Central	Central	South
**Copepoda**				
*Clausocalanus brevipes*	1	1	1	0
*Pareucalanus langae*	1	1	1	0
*Calocalanus* sp.	1	1	0	0
*Calocalanus contractus*	1	1	0	0
*Calocalanus pavo*	1	1	0	0
*Candacia catula*	1	1	0	0
*Corycaeus furcifer*	1	1	0	0
*Lucicutia flavicornis*	1	1	0	0
*Neocalanus gracilis*	1	1	0	0
*Phaenna spinifera*	1	1	0	0
*Pleuromamma gracilis*	1	1	0	0
*Pleuromamma quadrungulata*	1	1	0	0
*Scolecithrix bradyi*	1	1	0	0
*Aetideus giesbrechti*	1	0	1	1
*Calanoides acutus*	1	0	1	1
*Haloptilus longicornis*	1	0	1	1
*Aetideus australis*	1	0	1	0
*Oithona nana*	1	0	1	0
*Lubbockia squillimana*	1	0	0	1
*Scolecithricella glacialis*	1	0	0	1
*Acartia longiremis*	1	0	0	0
*Aegistus dubius*	1	0	0	0
*Aetideopsis carinata*	1	0	0	0
*Aetideus acutus*	1	0	0	0
*Calocalanus styliremis*	1	0	0	0
*Calocalanus tenuis*	1	0	0	0
*Candacia* sp.	1	0	0	0
*Candacia varicans*	1	0	0	0
*Centropages bradyi*	1	0	0	0
*Centropages gracilis*	1	0	0	0
*Centropages violaceus*	1	0	0	0
*Chiridius gracilis*	1	0	0	0
*Chirundina streetsii*	1	0	0	0
*Clausocalanus arcuicornis*	1	0	0	0
*Clausocalanus pergens*	1	0	0	0
*Clausocalanus* sp.	1	0	0	0
*Corycaeus giesbrechti*	1	0	0	0
*Corycaeus limbatus*	1	0	0	0
*Corycaeus speciosus*	1	0	0	0
*Ctenocalanus vanus*	1	0	0	0
*Eucalanus elongatus elongatus*	1	0	0	0
*Eucalanus hyalinus*	1	0	0	0
*Euchaeta acuta*	1	0	0	0
*Euchirella intermedia*	1	0	0	0
*Euchirella* sp.	1	0	0	0
*Farranula rostrata*	1	0	0	0
*Gaetanus miles*	1	0	0	0
*Gaetanus minor*	1	0	0	0
*Haloptilus spiniceps*	1	0	0	0
*Heterorhabdus robustus*	1	0	0	0
*Heterostylites major*	1	0	0	0
*Lophothrix latipes*	1	0	0	0
*Lucicutia clausi*	1	0	0	0
*Lucicutia curta*	1	0	0	0
*Lucicutia ovalis*	1	0	0	0
*Macrosetella gracilis*	1	0	0	0
*Mesocalanus tenuicornis*	1	0	0	0
*Nannocalanus minor*	1	0	0	0
*Nullosetigera helgae*	1	0	0	0
*Oculosetella gracilis*	1	0	0	0
*Oncaea* sp.	1	0	0	0
*Onychocorycaeus giesbrechti*	1	0	0	0
*Paracalanus parvus parvus*	1	0	0	0
*Paraheterorhabdus robustus*	1	0	0	0
*Pareuchaeta bisinuata*	1	0	0	0
*Pareuchaeta* sp.	1	0	0	0
*Pareuchaeta tonsa*	1	0	0	0
*Rhincalanus nasutus*	1	0	0	0
*Sapphirina* sp.	1	0	0	0
*Sapphirina angusta*	1	0	0	0
*Scaphocalanus* sp.	1	0	0	0
*Scolecithrix danae*	1	0	0	0
*Scottocalanus persecans*	1	0	0	0
*Scottocalanus securifrons*	1	0	0	0
*Spinocalanus brevicaudatus*	1	0	0	0
*Subeucalanus longiceps*	1	0	0	0
*Subeucalanus monachus*	1	0	0	0
*Undinula vulgaris*	1	0	0	0
*Valdiviella minor*	1	0	0	0
*Candacia maxima*	0	1	1	1
*Clausocalanus laticeps*	0	1	1	1
*Clytemnestra rostrata*	0	1	1	1
*Heterorhabdus papilliger*	0	1	1	1
*Pareuchaeta biloba*	0	1	1	0
*Candacia cheirura*	0	1	0	0
*Pareuchaeta gracilis*	0	1	0	0
*Pleuromamma robusta*	0	1	0	0
*Metridia gerlachei*	0	0	1	1
*Euchirella truncata*	0	0	1	0
*Gaetanus robustus*	0	0	1	0
*Lucicutia lucida*	0	0	1	0
*Mormonilla minor*	0	0	1	0
*Pareuchaeta sarsi*	0	0	1	0
*Undeuchaeta plumosa*	0	0	1	0
*Aetideopsis rostrata*	0	0	0	1
*Clausocalanus furcatus*	0	0	0	1
*Haloptilus ocellatus*	0	0	0	1
*Heterorhabdus compactus*	0	0	0	1
*Lubbockia aculeata*	0	0	0	1
**Amphipoda**				
*Phronima* sp.	1	1	1	0
*Cyphocaris* sp.	1	1	0	0
*Eupronoe* sp.	1	0	0	0
*Rhabdosoma* sp.	1	0	0	0
*Streetsia* sp.	1	0	0	0
*Vibilia* sp.	1	0	0	0
*Themisto* sp.	0	0	0	1
**Bivalvia larvae**	1	0	1	0
**Bryozoa larvae**	1	0	0	0
**Cephalopoda larvae**	1	0	0	0
**Decapoda larvae**	1	1	1	0
**Echinodermata larvae**	1	0	0	1
**Gastropoda larvae**	1	0	0	1
**Mysidacea** *Echinomysis*	0	0	0	1
**Polychaeta larvae**	1	1	0	0
**Salpidae**	1	0	1	0

**Note:**

Taxa sorted by major taxonomic groups then by presence in North assemblage, etc.

**Table 6:** List of taxa contributing to differences between mesoplankton assemblages bounded by four hydrological fronts, quantitative approach based on proportion of taxa (retrieved in Figs. 6–7).

Species	Average abundance				Average dissimilarity	Dissimilarity/ SD	Contribution (%)
	North	North-Central	Central	South			
**Copepoda**							
*Oithona similis*	148.67	646.95	303.15	915.04	26.71	1.66	39.87
Copepoda copepodites	43.81	220.11	89.41	200.1	7.21	1.51	10.86
*Ctenocalanus citer*	0.05	48.04	50.08	94.35	3.35	1.22	4.98
*Microcalanus pusillus*	25.12	69.89	5.31	37.07	2.85	0.90	4.28
*Triconia antarctica* ♂	0.27	2.52	7.11	46.85	1.24	1.28	1.91
*Clausocalanus brevipes*	11.34	32.89	11.62	0	1.09	1.20	1.64
*Clausocalanus pergens*	27.47	0	0	0	1.04	1.00	1.40
*Paracalanus parvus parvus*	21.77	0	0	0	0.87	0.69	1.17
*Pleuromamma gracilis*	21.79	0.97	0	0	0.83	0.83	1.12
*Oncaea* sp.	24.44	0	0	0	0.80	0.75	1.08
*Calanus propinquus*	0.71	16.94	0.03	1.64	0.62	0.74	0.96
**Euphausiacea** nauplii	2.62	82.24	36.99	88.67	3.31	1.10	4.99
**Appendicularia**	2.34	121.91	21.64	9.58	2.82	0.79	4.36
**Foraminifera**	10.2	38.57	27.87	107.36	2.81	1.30	4.24
**Radiolaria**	0.29	65.92	13.1	23.04	1.65	0.77	2.56

**Note:**

SIMPER analysis, species are arranged according major groups and further to their mean contribution. The list is cut at a 1% of contribution.

## Discussion

### Composition of zooplankton assemblages and distribution of dominant taxa

The cyclopoid *O. similis* was the most abundant species composing nearly half of the total mesoplankton abundance, which is likely explained by the time period surveyed (late spring-early summer), which is when the abundance of this species is maximal (Hosie et al., 2014). A high abundance of *O. similis* was previously recorded during the same time period (Stupnikova et al., 2018; Vedenin et al., 2019; Żmijevska, 1987), whereas in other seasons the contribution of this species was significantly lower (e.g., Atkinson & Peck, 1988; Pakhomov et al., 2000; Pakhomov & McQuaid, 1996; Takahashi et al., 2010). Other abundant taxa such as unidentified young calanoid copepodites, euphausiid nauplii, *Ctenocalanus citer*, and Foraminifera, were dominant in our samples and are known to be common in the Southern Ocean (Voronina, 1984; Vervoort, 1965; Bradford, 1969, 1971). Overall, the abundance of dominant taxa and biodiversity were distributed relatively homogenously across the ACC region except a plankton-depleted area at ~49° S.

The list of taxa identified in our samples aligns with previous records from the Southern Ocean (Mackintosh, 1937; Pakhomov et al., 2000, Pakhomov & Froneman, 2004a, 2004b; Froneman, 2000; Stupnikova & Vereshchaka, 2013; Voronina, 1984). The list is expected to include most mesoplankton taxa occurring in the Southern Ocean because seasonal upward migrations had generally occurred by the time of our survey (austral summer) (Hardy & Gunther, 1935; Mackintosh, 1934; Voronina, 1984; Rudjakov & Voronina, 1973) and mesoplankton typically concentrate in the sampled 0–300 m layer to feed and to reproduce (Gliwicz, 1986; Żmijevska, 1987; Lampert, 1989; Park & Ferrari, 2009).

Surprisingly, composition of zooplankton assemblages and distribution of dominant taxa remained relatively stable during at least 17 years. In fact, our results generally align with the data from the same transect in the late austral summer of 1992/1993 (Pakhomov et al., 2000). Variations in abundance, distribution, taxa richness, and bounding effects of the basic hydrological fronts are similar, which suggests a long-term stability of the basic mesoplankton characteristics along this transect. The surveys in 1993 and 2009 mainly differed in the sampling method: Bongo net and a single 0–300 m sample per station in 1993 and Judey net and three discrete layers per station in 2009. In addition, seasons were slightly different (December in 2009 vs. January–February in 1993). In 2009, the total abundance of mesoplankton in discrete layers along the transect ranged from 0.2 ind. m^−3^ to 13,743.6 ind. m^−3^ (five orders of magnitude), maximal and minimal values were recorded in the upper mixed and in the deepest layer, respectively. Within the combined 300-m layer, averaged abundances ranged from 16 to 1,455 ind. m^–3^ (two orders of magnitude). Consequently, total 0–300 m samples provide little information about actual variations of mesoplankton abundances within this layer.

In 1993, values for the same 0–300 m layer were one order of magnitude lower than in 2009 but variations were same (two orders of magnitude), from 2.1 to 211.5 ind. m^−3^. Judey nets are known to provide an order of magnitude higher mesoplankton abundance than Bongo nets (Voronina, 1984; Fransz & Gonzalez, 1997) and comparison of both surveys confirmed this finding. Maximal difference between surveys of 2009 (Judey net with mesh 0.18 mm) and 1993 (Bongo net with mesh 0.30 mm) was observed in the smallest fraction (*Oithona*, *Oncaea*, etc.), which likely passed through coarser Bongo mesh. Interestingly, the range of abundances, i.e., two orders of magnitude, remains constant regardless the net type and thus may be characteristic for mesoplankton of the Southern Ocean.

Both surveys showed similar local enrichments of mesoplankton abundance near the PF and the SAF, but differed in other patterns. A local peak north of the STF associated with the Agulhas Retroflection Current and found in 1993 was missing in 2009. Instead, local enrichments were recorded south and north of the STF, and were merely associated with mesoscale variability of the STF (Figs. 2A, 3A).

The next local peak near the northernmost position of the zero isotherm was narrow in 1993 and expanded over a wider latitudinal range (52° S–55.5° S) in 2009 (Fig. 3A here). This difference was likely induced by a greater advection of cold waters in 2009 (earlier survey season) relative to 1993.

Surprisingly, the survey in 2009 yielded nearly the same number of taxa as in 1993 (162 taxa in 1993 vs. 163 taxa in 2009). Thus, even significant difference in mesh size and net opening did not affect qualitative composition of mesoplankton. Conversely, different nets resulted in greatly different QNT-structure and the lists of dominant taxa. Among 15 most abundant taxa recorded in 1993 and 2009 (Table 2 here), only five taxa are common: *Oithona* sp., *Oncaea* sp., *Ctenocalanus* sp., *Clausocalanus* sp., and *Pleuromamma* sp. Like in 1993, taxa richness for the majority of stations varied between 15 and 25 taxa per station (Fig. 2E here). Pakhomov et al. (2000) indicated “slightly elevated number of taxa associated with … APF, SAF, STC and Agulhas Retroflection Current” but their Fig. 4A does not support this conclusion. Like Fig. 4A of Pakhomov et al. (2000), our Fig. 2E does not show any significant increase of taxa richness near any front or jet. The only visible trend is associated with an increase of taxa richness north of the STF (Fig. 2E) owing to a diversity of small subtropical copepods in mesoscale eddies of the Agulhas Current (compare Figs. 2A, 2B and Fig. 2E). Shannon (*H*) and Dominance (*D*) indices in 2009 (Figs. 2C, 2D) also do not show any visible trends along the transect (but show increase in biodiversity with depth). Regrettably, these indices were not presented in Pakhomov et al. (2000).

In both surveys, the basic separation of stations according to taxonomic composition of zooplankton in the whole layer 0–300 m coincided with the position of the STF, the SAF, and the PF. Despite the use of different sampling methods in 1993 and 2009, this basic grouping of plankton assemblages was not biased. As in 2009, secondary clusters were observed in 1993, but they are difficult to interpret because some stations were excluded from Fig. 6 of Pakhomov et al. (2000).

Overall, comparison of both surveys shows the existence of similar and stable plankton assemblages along the ST02 transect during a period 1992–2009. Similar spatial variations in abundance, distribution, taxa richness, and bounding effects of the basic hydrological fronts were found in both surveys regardless the sampling gear. Observed differences may be referred to sampling gear and temporal hydrological variability along the transect. In spite of observed spatio-temporal variability of mesoplankton, three principal hydrological fronts (the STF, the SAF, and the PF) represent clear biogeographic boundaries, which are detected on various size groups sampled by different gears.

### Comparative impact of major environmental factors on mesoplankton abundance and biodiversity

Although zooplankton assemblages in the Southern Ocean are influenced by various environmental factors, including temperature, ocean acidification, depth, thickness of sea ice, etc. (e.g., reviewed by Constable et al., 2014), the most notable factors are believed to be hydrological fronts and depth (Pakhomov, Perissinotto & McQuaid, 1994, Pakhomov et al., 2000; Pakhomov & Froneman, 2004a, 2004b; Froneman, 2000; Vedenin et al., 2019, 2020; Pakhomov & McQuaid, 1996; Pinkerton et al., 2002; Smetacek et al., 2002). Sea ice and climate indices may also be notable drivers but their impact have been so far recorded close to the Antarctic continent (Steinberg et al., 2015; Thibodeau et al., 2019). Our analyses of abundance, biodiversity and QUAL-structure of mesoplankton in the Southern Ocean transect SR02 confirm the importance of hydrological factors and sampled depth and provide a deeper insight into the hierarchy of environmental factors affecting these characteristics.

Our results indicate that depth greatly influences abundance, biodiversity, and QNT-structure of mesoplankton. Dominant taxa aggregate mostly in the upper mixed layer (Figs. 3B–3F); as a result, total plankton abundance (Fig. 3A) is also highest near the surface. This distribution mirrors vertical profiles of primary production, which is highest in the upper layer and decreases with the depth (Demidov, Mosharov & Gagarin, 2012). The vertical distribution of biodiversity indices has an opposite trend and increases with depth (Figs. 2C–2E). The upper mixed layer is dominated by few abundant taxa (Figs. 3B–3F), while the deepest layer is inhabited by a greater number of taxa more equally represented, which results in an increase of Shannon (*H*) and in a decrease of Dominance (*D*) indices with depth (Figs. 2C, 2D). Being a driver of abundance and biodiversity, depth greatly influences a QNT-structure of planktonic assemblages, but only slightly affects QUAL-structure depending on presence/absence of taxa (in the 0–300 m layer most taxa are recorded at all depths).

Hydrological zone strongly affects QUAL-structure and QNT-structure of mesoplankton assemblages. When we analyze QUAL-structure in CCAs (Figs. 4B, 4D) and ANOSIM tests (Table 2), hydrological zone becomes a dominant factor, regardless of the zone separation method (front or jets). A specific combination of environmental factors, such as temperature, salinity, etc., associated with each individual hydrological zone (water mass), affects QUAL-structure more than any other factor (the effect of depth is negligible in this case because most taxa occur throughout all sampled depth, although in different numbers). The effect of hydrological zone on QNT-structure of assemblages (linked to taxa proportions in samples) is less remarkable and comparable with the effect of depth (Figs. 4A, 4C).

Hydrological zone does not appear to notably affect distribution of mesoplankton abundance and biodiversity within the ACC area. The increase of abundance at 40° S (total abundance, abundance of *O. similis* and calanoid copepodites–Figs. 2A–2C) is likely linked to an anticyclonic eddy (part of the Agulhas meander), which incorporated Subantarctic waters of the ACC origin (details in Tarakanov & Gritsenko, 2014). A striking decrease in mesoplankton abundance around 49° S, especially prominent in the intermediate and deep layers (Figs. 3A–3E), is likely associated with a local increase of temperature and decrease of salinity in the same layers (Figs. 2A, 2B) and with the presence of a mesoscale warm core eddy. This eddy was recorded only in the intermediate and deepest layers, not in the upper mixed layer and not on a satellite image (we show the conditional position of this eddy in Fig. 1). The subsurface eddy was not considered by Tarakanov & Gritsenko (2014), its origin and genesis are unclear. Anyway, local effect of this eddy is visible in distribution of all mesoplankton characteristics and also mirrored in two stations, which greatly differed in QUAL- and QNT-structure from all recorded assemblages (black dots in Fig. 6). Interestingly, *Chl* measured in situ (Demidov, Mosharov & Gagarin, 2012) was also minimal within this zone. We only may suppose that the decrease of mesoplankton abundance may be related to a long-living eddy and to a consequent advection of deep plankton-depleted waters. Like abundances, biodiversity indices are more or less similar across various hydrological zones, except the northern stations within the core of the Agulhas Current (Indian Ocean species added) and the plankton-depleted area around 49° S discussed above. Thus, we did not find any significant effect of continuous hydrological fronts on biodiversity indices, even near the PF.

In this paper, we first tested productivity (*Chl*) as a possible driver of mesoplankton composition in the Southern Ocean. We understand that satellite *Chl* estimations are not ideal due to the lack of inclusion of sub-surface maxima and imperfection of algorithm calculations, which may lead to biased values (Garcia, Garcia & McClain, 2005; Zeng, Xu & Fischer, 2016; Brewin et al., 2017). However, potential impacts of productivity on integral characteristics of zooplankton could be identified by using *Chl* as a proxy of phytoplankton productivity and biomass (Vereshchaka et al., 2016, 2017; Vereshchaka, Lunina & Sutton, 2019). In our dataset, *Chl* is the third factor (after hydrological zone and depth) influencing the QNT-structure (Figs. 4A, 4C). Mesoplankton ascend to the surface for feeding and reproduction (Gliwicz, 1986; Żmijevska, 1987; Lampert, 1989; Park & Ferrari, 2009). As the amount of food affects abundance rather than presence/absence of taxa, QNT-structure of mesoplankton is much more dependent on *Chl* than QUAL-structure. In fact, along the OX axis explaining the main part of variance, the *Chl* vector is 0.28 % of the Zone-vector length under the quantitative approach (Fig. 4C) and only 0.16% of the Zone-vector length under the qualitative approach.

The fourth analyzed factor, diel cycle, is nearly negligible in CCAs (Figs. 4A–4D) and not statistically significant in the ANOSIM tests (Table 2), meaning day and night samples do not differ and diel migrations are not definite in our dataset. These results are in accordance with data of Pakhomov et al., 2000, which did not find significant differences in zooplankton densities between samples collected during the daytime and nighttime (*P* > 0.05). Studies on *Calanus acutus* (Hardy & Gunther, 1935) and *C. propinquus* (Mackintosh, 1934; Voronina, 1984; Rudjakov & Voronina, 1973) did not record diel migrations in the summer period as well. However our data do not suggest a complete absence of diel migrations for mesoplankton. More robust datasets (Vedenin et al., 2020) or other methods with greater vertical resolution (Vinogradov et al., 1996; Conroy et al., 2020) confirm diel migrations of low amplitude during the polar summer. Interestingly, even our dataset provides some evidence for diel migrations. In all CCAs plots (Figs. 4A–4D), diel cycle is opposed to depth (i.e., many taxa tend to occur at a lesser depth at night). While this effect is present in our study, it is not statistically significant, which suggests that diel cycle is the weakest among all of the analyzed factors.

### Comparative impact of hydrological fronts and jets on mesoplankton abundance and biodiversity

The hydrological fronts have varying effects on the mesoplankton. The STF divides subtropical and subpolar fauna and thus has the strongest impact. The vicinities of the STF were previously recognized as a transitional zone between the Subantarctic and the Subtropical Zones (McGinnis, 1977; Lomakina, 1964) with eddies and meanders causing local discontinuities in species distribution, which are also visible in our dataset (Fig. 5, north of the STF). The dominating impact of the STF is confirmed by a statistically significant difference between assemblages north and south of this front (Tables 3–4, Appendices 3, 5) and by clustering (least similarity in Figs. 6–7, Appendices 7–9).

The PF is the second strongest front: discontinuity of plankton assemblages is distinct (NUT–Fig. 5) and the division of neighboring assemblages is statistically robust (Table 3, Appendix 3) at all depths. However, assemblages on both sides of the PF are more similar than those on both sides of the STF (Figs. 6–7, Appendices 7–9).

The effect of the SAF is weaker than that of the STF and the PF, which may explain a current uncertainty in the literature. Indeed, the impact of the STF and the PF on plankton distribution in the Southern Ocean has been recognized for a long time (Foxton, 1956; Hopkins, 1971; Pakhomov & McQuaid, 1996; Dolzhenkov, 1982; Deacon, 1982; Grachev, 1991), whereas the role of the SAF was less certain (Pakhomov & McQuaid, 1996; Pakhomov et al., 2000). A possible explanation is that the effect of the SAF may differ depending on geographic area and/or season. In the Drake Passage, the SAF and the PF may have a subequal effect (Hosie et al., 2014; Vedenin et al., 2019), whereas in our survey the SAF does not affect distribution in the deep layer (the PF influences all sampled depths: Appendix 3, Table 3) and does not provide significant faunal discontinuity (Fig. 5).

The SB is the weakest hydrological front affecting mesoplankton only in the intermediate layer (Fig. 5, Table 3). This particular influence may be explained by an advection from the cold waters of the Weddell Sea, which exhibited negative temperature values in the intermediate layer in the time period of this survey (Fig. 2A).

Overall, we observed the following hierarchy of hydrological fronts: the STF, the PF, the SAF, and the SB. The effect of the first three fronts on plankton assemblages is strong and decreases with the depth (Table 3).

The effect of the dynamic jets, which are not associated with hydrological fronts, is less regular and weaker than that of hydrological fronts. For example, two jets of the SACCF divided plankton assemblages in the intermediate layer (SACCF-N) or in the upper and deep layers (SACCF-S) (Table 4, Appendix 5). A possible explanation of this result may be related to an intrusion of cold waters from the south: the isotherm of +1 °C located in the SACCF-S in the upper mixed layer moved north and crossed SACCF-N in the intermediate layer and then moved back south to cross the SACCF-S in the deep layer (Fig. 2A). It is important to note that local eddies and meanders of various origin and evolution (Tarakanov & Gritsenko, 2014) may strongly affect biodiversity (Figs. 2C–2E), abundance (Fig. 3A–3F) and QUAL-structure of mesoplankton (Fig. 5). These hydrological structures may be either enriched (cyclonic gyre of the Agulhas Current north of 36° S) or depleted in mesoplankton (a warm core eddy around 49° S).

Overall, analyses of discontinuities (Fig. 5), ANOSIM tests (Tables 3, 4, Appendices 3, 5) and cluster analyses (Figs. 6–7, Appendices 7–9), all provided evidence for a strong effect of hydrological fronts and uncertain effect of dynamic jets. This result suggests that mesoplankton composition is driven by hydrological parameters and further maintained through compartmentalization by hydrological fronts.

Differences in QUAL- and QNT-structure of mesoplankton assemblages bounded by hydrological fronts are mainly explained by copepods (Tables 5–6). This is not surprising, because copepods are the most diverse and most abundant group defining the face of the mesoplankton in the Southern Ocean. Among copepods, *Oithona similis* is the most important species explaining nearly 40% of variance in QNT-structure of mesoplankton in different hydrological zones.

### Concluding Remarks

Combining all three vertical strata and analyzing plankton assemblages within the whole 0–300 m layer (Fig. 6 vs. Appendices 7–9) provides a more sound output, as the dataset in this case is more representative and not biased by fluctuations in vertical distribution of taxa. At the same time, integral samples provide little information about actual variations of mesoplankton abundances. In our dataset, mesoplankton abundances vary by five orders of magnitude in discrete vertical samples and “only” two orders of magnitude in combined 0–300 m samples.

QUAL-structure (presence or absence of taxa) of mesoplankton assemblages precisely mirrors hydrological structure: all recorded assemblages are definitely bounded by hydrological fronts. QUAL-structure is only slightly variable in time and space and may be recommended for future analyses of plankton biogeography of dynamic areas, such as the Southern Ocean. The only disadvantage of this method is related to a time-consuming identification, because ideally all taxa need to be identified to a lowest possible level (in our dataset 163 taxa). Each taxon, either rare or dominant, equally contributes to the QUAL-structure; therefore inaccurate or undetailed identification may greatly bias results. Although time-consuming, qualitative-structure may be recommended for successful biogeographic analyses of the Southern Ocean. Conversely, the quantitative approach is less sensitive to a quality of rare taxa identification and therefore less time consuming (only dominant and subdominant taxa may be identified). At the same time, QNT-structure strongly depends on proportion of taxa, which, in turn, depends on various factors, both current and precedent. Among these factors are the dynamics of particular water masses, local food resources and carnivores, life cycles in particular locations, interaction between species, etc., which may mask proper biogeographic boundaries.

**Figure 9:**
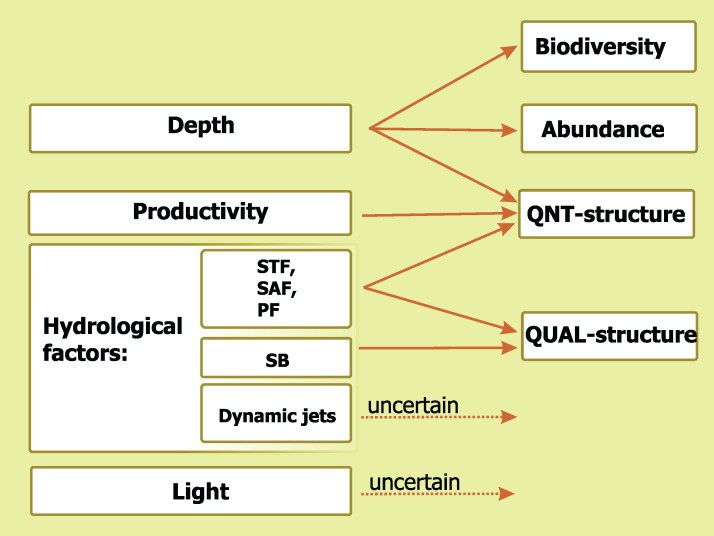
Concluding scheme showing the relative impact of major environmental factors and individual hydrological structures on general characteristics of plankton assemblages (white). Hydrological front coding: Subtropical Front (STF), Subantarctic Front (SAF), Polar Front (PF), and Southern Boundary (SB).

A comparative impact of the analyzed environmental factors is summarized in Fig. 9. Depth influences three basic mesoplankton characteristics, hydrological factors drives two of them, *Chl* strongly affects one parameter, and diel cycle does not significantly influence any of the characteristics. The STF, the PF, and the SAF drive QNT-structure and QUAL-structure of mesoplankton (in decreasing order of influence), while the SB affects only QUAL-structure.

The basic outcome of this paper is a hierarchy of environmental factors affecting mesoplankton distribution and biodiversity. Our results show that mesoplankton composition is driven by hydrological parameters and further maintained through compartmentalization by fronts. Dynamic jets not associated with hydrological fronts have no significant effect on mesoplankton biodiversity and abundance and their position may be discarded in future hydrobiological surveys. Although characterized by two-fold fluctuations in abundances, mesoplankton distribution did not significantly change during the period 1992–2009 in the core Southern Ocean. This surprising result contrasts with recent data on climate change effect on coastal plankton assemblages of the Southern Ocean and merits new supporting/rejecting data and future surveys.

## Supplemental Information

10.7717/peerj.11411/supp-1 Supplemental Information 1 Sample list. Average temperature and salinity values are provided for layers sampled (Temperature Av and Salinity Av): a depth of 10 m (Temperature Surface and Salinity Surface), and a depth of 300 m (deepest sampled horizon), Temperature 300 m. Sampled layers: upper mixed (1), intermediate (2), and deep (3). Hydrological Zones: north of STF (1), between STF and SAF-N (2), between SAF-N and SAF-M (3), between SAF-M and SAF-S (4), between SAF-S and PF (5), between PF and SACCF-N (6), between SACCF-N and SACCF-S (7), between SACCF-S and SB (8), and south of SB (9).

10.7717/peerj.11411/supp-2 Supplemental Information 2 Full list of taxa ordered according to their percent contribution to the total mesoplankton abundance.

10.7717/peerj.11411/supp-3 Supplemental Information 3 Results of the one-way ANOSIM analyses with zones bounded by four hydrological fronts as a grouping factor for the upper mixed layer (A), intermediate layer (B), deep layer (C), and whole 0-300 m layer. Hydrological front coding (vertical black lines): Subtropical Front (STF); Subantarctic Front (SAF-M), Polar Front (PF), Southern Boundary (SB). Solid and dotted lines indicate statistically significant and insignificant boundaries, respectively.

10.7717/peerj.11411/supp-4 Supplemental Information 4 Results of ANOSIM tests used to indicate the difference between plankton assemblages bounded by hydrological fronts (Bray-Curtis quantitative index used). Tested layers: upper mixed (Layer 1), intermediate (Layer 2), deep (Layer 3), and whole 0-300 m layer (Layer 0). Zones: north of STF (1), between STF and SAF-M (2), between SAF-M and PF (3), between PF and SB (4), and south of SB (5). Statistically significant boundaries between neighburing zones are in bold.

10.7717/peerj.11411/supp-5 Supplemental Information 5 Results of the one-way ANOSIM analyses with zones bounded by eight dynamic jets as a grouping factor for the upper mixed layer (A), intermediate layer (B), deep layer (C), and the whole 0-300 m layer. Hydrological jet coding (vertical black lines): Subtropical Front (STF); Subantarctic Front (SAF), three branches (northern SAF-N, middle SAF-M and southern SAF-S); Polar Front (PF), merged branches; Southern Front (SACCF), two branches (northern SACCF-N and southern SACCF-S); Southern Boundary (SB). Solid and dotted lines indicate statistically significant and insignificant boundaries, respectively.

10.7717/peerj.11411/supp-6 Supplemental Information 6 Results of the ANOSIM tests determining the difference between plankton asseblages bounded by dynamic jets (Bray-Curtis quantitative index). Tested layers: upper mixed (Layer 1), intermediate (Layer 2), deep (Layer 3), and the entire 0-300 m layer (Layer 0). Zones: north of STF (1), between STF and SAF-N (2), between SAF-N and SAF-M (3), between SAF-M and SAF-S (4), between SAF-S and PF (5), between PF and SACCF-N (6), between SACCF-N and SACCF-S (7), between SACCF-S and (8), and south of SB (9). Statistically significant boundaries between neighboring zones are in bold.

10.7717/peerj.11411/supp-7 Supplemental Information 7 Results from the cluster analysis (Bray-Curtis qualitative index) for samples collected within the upper mixed layer. All clades and nodes are robust (p <0.05), and red lines indicate minimal similarity between two marked clades (exact value unrecognizable). Colored figures on bottom indicate robust clusters.

10.7717/peerj.11411/supp-8 Supplemental Information 8 Results from the cluster analysis (Bray-Curtis qualitative index) for samples collected within the intermediate layer. Colored figures on bottom indicate robust clusters.

10.7717/peerj.11411/supp-9 Supplemental Information 9 Results from the cluster analysis (Bray-Curtis qualitative index) for samples collected within the deep layer. Colored figures on bottom indicate robust clusters.

10.7717/peerj.11411/supp-10 Supplemental Information 10 Results from the cluster analysis (Bray-Curtis quantitative similarity index) from samples collected within the upper mixed layer (Layer 1) and intermediate layer (Layer 2). Colors indicate groups diverging at certain similarity levels.

10.7717/peerj.11411/supp-11 Supplemental Information 11 Results from the cluster analysis (Bray-Curtis quantitative similarity index) for samples collected within the deep layer (Layer 3) and entire 0-300 m layer (Layer 0). Colors indicate groups diverging at certain similarity levels.

10.7717/peerj.11411/supp-12 Supplemental Information 12 Raw data: Coordinates of stations, biomass measurements, temperature and salinity data.

## Additional Information and Declarations

### Field Study Permissions

The following information was supplied relating to field study approvals (i.e., approving body and any reference numbers): Field works and sample collection were performed in the open ocean out of the Exclusive Economic Zones and no need any special permission or approval.

### Data Availability

The following information was supplied regarding data availability: Coordinates of stations, biomass measurements, temperature & salinity data, and MDS data are available in the Supplemental File.
