# Supplementary material for: Biogeography of the Southern Ocean: environmental factors driving mesoplankton distribution South of Africa
Source: PeerJ. 2021 May 10;9:e11411. doi: 10.7717/peerj.11411 (PMC8117931; doi:10.7717/peerj.11411)
Supplement: Supplemental Information 1 — Sampled layers: upper mixed (1), intermediate (2), and deep (3). Hydrological Zones: north of STF (1), between STF and SAF-N (2), between SAF-N and SAF-M (3), between SAF-M and SAF-S (4), between SAF-S and PF (5), between PF and SACCF-N (6), between SACCF-N and SACCF-S (7), between SACCF-S and SB (8), and south of SB (9). [file peerj-09-11411-s001.docx]

Appendix 1. Sample list. Average temperature and salinity values are provided for layers sampled (Temperature Av and Salinity Av): a depth of 10 m (Temperature Surface and Salinity Surface), and a depth of 300 m (deepest sampled horizon, Temperature 300 m and Salinity 300 m). Sampled layers: upper mixed (1), intermediate (2), and deep (3). Hydrological Zones: north of STF (1), between STF and SAF-N (2), between SAF-N and SAF-M (3), between SAF-M and SAF-S (4), between SAF-S and PF (5), between PF and SACCF-N (6), between SACCF-N and SACCF-S (7), between SACCF-S and SB (8), and south of SB (9).

| **Station** | **Date** | **Time** | **Latitude, º S** | **Longitude,º E** | **Temperature Av, C°** | **Temperature Surface, C°** | **Temperature 300 m, C°** | **Salinity Av** | **Salinity Surface** | **Salinity 300 m** | **Depth, m** | **Sampled layer, m** | **Volume filtered, m^3^** | **Layer** | **Hydrological Zone** |
| --- | --- | --- | --- | --- | --- | --- | --- | --- | --- | --- | --- | --- | --- | --- | --- |
| 2206 | 04.12.2009 | 8:40 | 34.4365 | 14.4093 E | 18.79 | 18.81 | 15.2 | 35.54 | 35.62 | 35.43 | 4507 | 0-54 | 5.4 | 1 | 1 |
| 2206 | 04.12.2009 | 8:30 | 34.4365 | 14.4093 E | 17.9 | 18.81 | 15.2 | 35.54 | 35.62 | 35.43 | 4507 | 44-160 | 11.6 | 2 | 1 |
| 2206 | 04.12.2009 | 8:15 | 34.4365 | 14.4093 E | 16.61 | 18.81 | 15.2 | 35.54 | 35.62 | 35.43 | 4507 | 160-286 | 12.6 | 3 | 1 |
| 2208 | 04.12.2009 | 21:35 | 35.02 | 14.0642 E | 19.26 | 19.28 | 13.57 | 35.58 | 35.58 | 35.18 | 4670 | 0-40 | 4 | 1 | 1 |
| 2208 | 04.12.2009 | 21:15 | 35.02 | 14.0642 | 17.18 | 19.28 | 13.57 | 35.53 | 35.58 | 35.18 | 4670 | 41-181 | 14 | 2 | 1 |
| 2208 | 04.12.2009 | 21:00 | 35.02 | 14.0642 | 15.11 | 19.28 | 13.57 | 35.4 | 35.58 | 35.18 | 4670 | 169-300 | 13.1 | 3 | 1 |
| 2210 | 05.12.2009 | 8:55 | 35.6248 | 13.6818 | 18.7 | 18.7 | 15.29 | 35.62 | 35.62 | 35.44 | 4472 | 0-41 | 4.1 | 1 | 1 |
| 2210 | 05.12.2009 | 8:45 | 35.6248 | 13.6818 | 18 | 18.7 | 15.29 | 35.59 | 35.62 | 35.44 | 4472 | 40-188 | 15.3 | 2 | 1 |
| 2210 | 05.12.2009 | 8:30 | 35.6248 | 13.6818 | 16.12 | 18.7 | 15.29 | 35.5 | 35.62 | 35.44 | 4472 | 188-299 | 11.1 | 3 | 1 |
| 2212 | 05.12.2009 | 22:57 | 36.239 | 13.308 | 18.65 | 18.65 | 14.81 | 35.62 | 35.62 | 35.4 | 4830 | 0-30 | 3 | 1 | 1 |
| 2212 | 05.12.2009 | 22:15 | 36.239 | 13.308 | 18.09 | 18.65 | 14.81 | 35.6 | 35.62 | 35.4 | 4830 | 30-125 | 10 | 2 | 1 |
| 2212 | 05.12.2009 | 22:25 | 36.239 | 13.308 | 16.39 | 18.65 | 14.81 | 35.53 | 35.62 | 35.4 | 4830 | 122-300 | 17.8 | 3 | 1 |
| 2214 | 06.12.2009 | 8:45 | 36.8322 | 12.9385 | 18.44 | 18.45 | 12.47 | 35.61 | 35.61 | 35.1 | 4959 | 0-41 | 4.1 | 1 | 1 |
| 2214 | 06.12.2009 | 8:35 | 36.8322 | 12.9385 | 16.15 | 18.45 | 12.47 | 35.5 | 35.61 | 35.1 | 4959 | 40-170 | 13 | 2 | 1 |
| 2214 | 06.12.2009 | 8:25 | 36.8322 | 12.9385 | 13.54 | 18.45 | 12.47 | 35.22 | 35.61 | 35.1 | 4959 | 165-310 | 14.5 | 3 | 1 |
| 2216 | 06.12.2009 | 21:25 | 37.4183 | 12.5513 | 17.37 | 17.39 | 11.42 | 35.59 | 35.6 | 35 | 5053 | 0-30 | 3 | 1 | 1 |
| 2216 | 06.12.2009 | 21:15 | 37.4183 | 12.5513 | 14.29 | 17.39 | 11.42 | 35.29 | 35.6 | 35 | 5053 | 30-212 | 18.2 | 2 | 1 |
| 2216 | 06.12.2009 | 21:00 | 37.4183 | 12.5513 | 11.69 | 17.39 | 11.42 | 34.95 | 35.6 | 35 | 5053 | 212-300 | 8.8 | 3 | 1 |
| 2218 | 07.12.2009 | 8:55 | 37.9893 | 12.1565 | 15.75 | 15.75 | 8.85 | 35.2 | 35.2 | 34.6 | 5172 | 0-35 | 3.5 | 1 | 1 |
| 2218 | 07.12.2009 | 8:40 | 37.9893 | 12.1565 | 11.85 | 15.75 | 8.85 | 34.87 | 35.2 | 34.6 | 5172 | 34-202 | 16.8 | 2 | 1 |
| 2218 | 07.12.2009 | 8:25 | 37.9893 | 12.1565 | 9.5 | 15.75 | 8.85 | 34.68 | 35.2 | 34.6 | 5172 | 202-310 | 10.8 | 3 | 1 |
| 2220 | 07.12.2009 | 18:50 | 38.5898 | 11.7487 | 16.23 | 16.33 | 9.25 | 35.44 | 35.44 | 34.7 | 5139 | 0-40 | 4 | 1 | 1 |
| 2220 | 07.12.2009 | 18:35 | 38.5898 | 11.7487 | 12.73 | 16.33 | 9.25 | 35.07 | 35.44 | 34.7 | 5139 | 40-200 | 16 | 2 | 1 |
| 2220 | 07.12.2009 | 18:15 | 38.5898 | 11.7487 | 10.13 | 16.33 | 9.25 | 34.8 | 35.44 | 34.7 | 5139 | 200-302 | 10.2 | 3 | 1 |
| 2222 | 08.12.2009 | 8:20 | 39.1843 | 11.3302 | 16.56 | 16.56 | 11.34 | 35.57 | 35.57 | 34.89 | 5130 | 0-60 | 6 | 1 | 1 |
| 2222 | 08.12.2009 | 8:45 | 39.1843 | 11.3302 | 14.55 | 16.56 | 11.34 | 35.28 | 35.57 | 34.89 | 5130 | 60-195 | 13.5 | 2 | 1 |
| 2222 | 08.12.2009 | 9:20 | 39.1843 | 11.3302 | 12.3 | 16.56 | 11.34 | 35.04 | 35.57 | 34.89 | 5130 | 203-300 | 9.7 | 3 | 1 |
| 2224 | 08.12.2009 | 18:00 | 39.6823 | 10.9302 | 16.98 | 17.09 | 14.99 | 35.6 | 35.6 | 35.41 | 4668 | 0-48 | 4.8 | 1 | 1 |
| 2224 | 08.12.2009 | 17:40 | 39.6823 | 10.9302 | 16.89 | 17.09 | 14.99 | 35.6 | 35.6 | 35.41 | 4668 | 48-190 | 14 | 2 | 1 |
| 2224 | 08.12.2009 | 17:50 | 39.6823 | 10.9302 | 16.09 | 17.09 | 14.99 | 35.54 | 35.6 | 35.41 | 4668 | 190-300 | 11 | 3 | 1 |
| 2226 | 09.12.2009 | 4:55 | 40.3775 | 10.532 | 16.79 | 16.87 | 13.15 | 35.6 | 35.6 | 35.19 | 4639 | 0-50 | 5 | 1 | 1 |
| 2226 | 09.12.2009 | 4:50 | 40.3775 | 10.532 | 16.7 | 16.87 | 13.15 | 35.59 | 35.6 | 35.19 | 4639 | 48-120 | 7.2 | 2 | 1 |
| 2226 | 09.12.2009 | 5:05 | 40.3775 | 10.532 | 14.46 | 16.87 | 13.15 | 35.33 | 35.6 | 35.19 | 4639 | 123-319 | 19.6 | 3 | 1 |
| 2228 | 09.12.2009 | 16:35 | 40.9778 | 10.0573 | 15.9 | 16.17 | 9.86 | 35.46 | 35.46 | 34.79 | 4559 | 0-78 | 7.8 | 1 | 1 |
| 2228 | 09.12.2009 | 16:25 | 40.9778 | 10.0573 | 13.31 | 16.17 | 9.86 | 35.16 | 35.46 | 34.79 | 4559 | 78-198 | 12 | 2 | 1 |
| 2228 | 09.12.2009 | 16:15 | 40.9778 | 10.0573 | 10.78 | 16.17 | 9.86 | 34.88 | 35.46 | 34.79 | 4559 | 199-300 | 10.1 | 3 | 1 |
| 2230 | 10.12.2009 | 2:45 | 41.5328 | 9.6432 | 16 | 16.04 | 9.4 | 35.45 | 35.43 | 34.73 | 4730 | 0-45 | 4.5 | 1 | 2 |
| 2230 | 10.12.2009 | 3:00 | 41.5328 | 9.6432 | 12 | 16.04 | 9.4 | 35.11 | 35.43 | 34.73 | 4730 | 42-155 | 11.3 | 2 | 2 |
| 2230 | 10.12.2009 | 2:50 | 41.5328 | 9.6432 | 10.22 | 16.04 | 9.4 | 34.8 | 35.43 | 34.73 | 4730 | 155-300 | 14.5 | 3 | 2 |
| 2232 | 10.12.2009 | 14:30 | 42.11 | 9.1905 | 10.4 | 11.67 | 7.22 | 34.43 | 34.47 | 34.41 | 4745 | 0-60 | 6 | 1 | 2 |
| 2232 | 10.12.2009 | 14:20 | 42.11 | 9.1905 | 8.2 | 11.67 | 7.22 | 34.39 | 34.47 | 34.41 | 4745 | 58-200 | 14.2 | 2 | 2 |
| 2232 | 10.12.2009 | 14:05 | 42.11 | 9.1905 | 7.49 | 11.67 | 7.22 | 34.4 | 34.47 | 34.41 | 4745 | 199-300 | 10.1 | 3 | 2 |
| 2234 | 11.12.2009 | 1:05 | 42.6912 | 8.7402 | 10.46 | 11.48 | 6.79 | 34.38 | 34.39 | 34.37 | 4903 | 0-50 | 5 | 1 | 2 |
| 2234 | 11.12.2009 | 0:55 | 42.6912 | 8.7402 | 8.21 | 11.48 | 6.79 | 34.39 | 34.39 | 34.37 | 4903 | 50-220 | 17 | 2 | 2 |
| 2234 | 11.12.2009 | 0:45 | 42.6912 | 8.7402 | 7.29 | 11.48 | 6.79 | 34.4 | 34.39 | 34.37 | 4903 | 218-300 | 8.2 | 3 | 2 |
| 2236 | 11.12.2009 | 12:20 | 43.2668 | 8.2662 | 9.92 | 11 | 7.61 | 34.38 | 34.4 | 34.43 | 2439 | 0-59 | 6.1 | 1 | 2 |
| 2236 | 11.12.2009 | 12:00 | 43.2668 | 8.2662 | 8.55 | 11 | 7.61 | 34.4 | 34.4 | 34.43 | 2439 | 58-120 | 6.2 | 2 | 2 |
| 2236 | 11.12.2009 | 12:10 | 43.2668 | 8.2662 | 7.92 | 11 | 7.61 | 34.42 | 34.4 | 34.43 | 2439 | 120-300 | 18.6 | 3 | 2 |
| 2238 | 11.12.2009 | 23:20 | 43.8445 | 7.7982 | 10.26 | 10.72 | 7.18 | 34.49 | 34.51 | 34.42 | 4451 | 0-61 | 6.1 | 1 | 3 |
| 2238 | 11.12.2009 | 23:10 | 43.8445 | 7.7982 | 9.04 | 10.72 | 7.18 | 34.54 | 34.51 | 34.42 | 4451 | 63-180 | 11.7 | 2 | 3 |
| 2238 | 11.12.2009 | 23:00 | 43.8445 | 7.7982 | 7.78 | 10.72 | 7.18 | 34.47 | 34.51 | 34.42 | 4451 | 185-300 | 11.5 | 3 | 3 |
| 2240 | 12.12.2009 | 12:10 | 44.4175 | 7.326 | 8.7 | 9.51 | 5.18 | 34.26 | 34.27 | 34.22 | 4577 | 0-81 | 8.1 | 1 | 3 |
| 2240 | 12.12.2009 | 12:00 | 44.4175 | 7.326 | 6.99 | 9.51 | 5.18 | 34.25 | 34.27 | 34.22 | 4577 | 80-173 | 9.3 | 2 | 3 |
| 2240 | 12.12.2009 | 11:50 | 44.4175 | 7.326 | 6.01 | 9.51 | 5.18 | 34.27 | 34.27 | 34.22 | 4577 | 173-300 | 12.7 | 3 | 3 |
| 2242 | 13.12.2009 | 0:15 | 44.9787 | 6.8065 | 7.46 | 7.63 | 4 | 33.86 | 33.84 | 34.14 | 4230 | 0-50 | 5 | 1 | 4 |
| 2242 | 13.12.2009 | 0:10 | 44.9787 | 6.8065 | 5.71 | 7.63 | 4 | 33.97 | 33.84 | 34.14 | 4230 | 50-145 | 9.5 | 2 | 4 |
| 2242 | 13.12.2009 | 0:00 | 44.9787 | 6.8065 | 4.52 | 7.63 | 4 | 34.1 | 33.84 | 34.14 | 4230 | 150-311 | 16.1 | 3 | 4 |
| 2244 | 13.12.2009 | 12:25 | 45.6285 | 6.1333 | 6.81 | 7.16 | 3.66 | 33.8 | 33.8 | 34.12 | 4664 | 0-50 | 5 | 1 | 4 |
| 2244 | 13.12.2009 | 12:15 | 45.6285 | 6.1333 | 4.76 | 7.16 | 3.66 | 33.84 | 33.8 | 34.12 | 4664 | 50-173 | 12.3 | 2 | 4 |
| 2244 | 13.12.2009 | 12:00 | 45.6285 | 6.1333 | 3.94 | 7.16 | 3.66 | 34.08 | 33.8 | 34.12 | 4664 | 173-300 | 12.7 | 3 | 4 |
| 2246 | 13.12.2009 | 23:25 | 46.0973 | 5.7627 | 7.14 | 7.4 | 3.44 | 33.74 | 33.77 | 34.11 | 4805 | 0-40 | 4 | 1 | 4 |
| 2246 | 13.12.2009 | 23:15 | 46.0973 | 5.7627 | 5.2 | 7.4 | 3.44 | 33.79 | 33.77 | 34.11 | 4805 | 40-170 | 13 | 2 | 4 |
| 2246 | 13.12.2009 | 23:00 | 46.0973 | 5.7627 | 3.82 | 7.4 | 3.44 | 34.05 | 33.77 | 34.11 | 4805 | 174-300 | 12.6 | 3 | 4 |
| 2247 | 14.12.2009 | 4:45 | 46.3857 | 5.5047 | 6.73 | 7 | 3.66 | 33.74 | 33.77 | 34.12 | 4375 | 0-60 | 6 | 1 | 4 |
| 2247 | 14.12.2009 | 4:35 | 46.3857 | 5.5047 | 4.61 | 7 | 3.66 | 33.81 | 33.77 | 34.12 | 4375 | 60-175 | 11.5 | 2 | 4 |
| 2247 | 14.12.2009 | 4:20 | 46.3857 | 5.5047 | 3.85 | 7 | 3.66 | 34.08 | 33.77 | 34.12 | 4375 | 175-300 | 12.5 | 3 | 4 |
| 2249 | 14.12.2009 | 16:20 | 46.9277 | 4.97 | 6.81 | 7.2 | 3.77 | 33.84 | 33.83 | 34.12 | 4181 | 0-58 | 5.8 | 1 | 4 |
| 2249 | 14.12.2009 | 16:10 | 46.9277 | 4.97 | 4.98 | 7.2 | 3.77 | 33.92 | 33.83 | 34.12 | 4181 | 58-204 | 14.6 | 2 | 4 |
| 2249 | 14.12.2009 | 15:50 | 46.9277 | 4.97 | 4.05 | 7.2 | 3.77 | 34.1 | 33.83 | 34.12 | 4181 | 204-300 | 9.6 | 3 | 4 |
| 2251 | 15.12.2009 | 5:00 | 47.4983 | 4.422 | 7.1 | 7.09 | 3.68 | 33.8 | 33.8 | 34.12 | 4470 | 0-40 | 4 | 1 | 4 |
| 2251 | 15.12.2009 | 4:25 | 47.4983 | 4.422 | 5.31 | 7.09 | 3.68 | 33.93 | 33.8 | 34.12 | 4470 | 40-200 | 16 | 2 | 4 |
| 2251 | 15.12.2009 | 4:30 | 47.4983 | 4.422 | 4.07 | 7.09 | 3.68 | 34.1 | 33.8 | 34.12 | 4470 | 200-305 | 10.5 | 3 | 4 |
| 2253 | 15.12.2009 | 18:00 | 48.0575 | 3.996 | 6.75 | 6.9 | 3.65 | 33.77 | 33.78 | 34.11 | 4391 | 0-72 | 7.2 | 1 | 4 |
| 2253 | 15.12.2009 | 18:10 | 48.0575 | 3.996 | 4.35 | 6.9 | 3.65 | 33.9 | 33.78 | 34.11 | 4391 | 72-225 | 15.3 | 2 | 4 |
| 2253 | 15.12.2009 | 18:20 | 48.0575 | 3.996 | 7.1 | 6.9 | 3.65 | 33.8 | 33.78 | 34.11 | 4391 | 225-306 | 8.1 | 3 | 4 |
| 2255 | 16.12.2009 | 8:50 | 48.5278 | 3.3028 | 6.06 | 6.07 | 3.02 | 33.76 | 33.78 | 34.12 | 4062 | 0-62 | 6.2 | 1 | 5 |
| 2255 | 16.12.2009 | 8:40 | 48.5278 | 3.3028 | 4.23 | 6.07 | 3.02 | 33.86 | 33.78 | 34.12 | 4062 | 60-182 | 12.2 | 2 | 5 |
| 2255 | 16.12.2009 | 8:30 | 48.5278 | 3.3028 | 3.28 | 6.07 | 3.02 | 34.09 | 33.78 | 34.12 | 4062 | 182-300 | 11.8 | 3 | 5 |
| 2257 | 16.12.2009 | 23:50 | 49.1713 | 2.6988 | 4.91 | 5.12 | 2.42 | 33.79 | 33.79 | 34.16 | 4052 | 0-80 | 8 | 1 | 5 |
| 2257 | 16.12.2009 | 23:30 | 49.1713 | 2.6988 | 3.11 | 5.12 | 2.42 | 33.89 | 33.79 | 34.16 | 4052 | 75-188 | 11.3 | 2 | 5 |
| 2257 | 16.12.2009 | 23:15 | 49.1713 | 2.6988 | 2.52 | 5.12 | 2.42 | 34.11 | 33.79 | 34.16 | 4052 | 188-315 | 13.2 | 3 | 5 |
| 2259 | 17.12.2009 | 15:10 | 49.6893 | 2.0833 | 5.02 | 5.03 | 2.35 | 33.79 | 33.79 | 34.17 | 3839 | 0-90 | 9 | 1 | 5 |
| 2259 | 17.12.2009 | 14:55 | 49.6893 | 2.0833 | 3.35 | 5.03 | 2.35 | 33.88 | 33.79 | 34.17 | 3839 | 95-192 | 9.7 | 2 | 5 |
| 2259 | 17.12.2009 | 14:40 | 49.6893 | 2.0833 | 2.31 | 5.03 | 2.35 | 34.09 | 33.79 | 34.17 | 3839 | 192-300 | 10.8 | 3 | 5 |
| 2261 | 18.12.2009 | 4:15 | 50.2323 | 1.4103 | 4.95 | 5 | 2.6 | 33.79 | 33.78 | 34.13 | 3035 | 0-100 | 10 | 1 | 5 |
| 2261 | 18.12.2009 | 4:10 | 50.2323 | 1.4103 | 3.47 | 5 | 2.6 | 33.86 | 33.78 | 34.13 | 3035 | 100-180 | 8 | 2 | 5 |
| 2261 | 18.12.2009 | 3:50 | 50.2323 | 1.4103 | 2.83 | 5 | 2.6 | 34.08 | 33.78 | 34.13 | 3035 | 182-300 | 11.8 | 3 | 5 |
| 2263 | 18.12.2009 | 16:23 | 50.7673 | 0.8265 | 4.02 | 4.25 | 2.21 | 33.81 | 33.81 | 34.25 | 3470 | 0-92 | 9.2 | 1 | 6 |
| 2263 | 18.12.2009 | 16:15 | 50.7673 | 0.8265 | 1.78 | 4.25 | 2.21 | 33.95 | 33.81 | 34.25 | 3470 | 91-195 | 10.4 | 2 | 6 |
| 2263 | 18.12.2009 | 15:55 | 50.7673 | 0.8265 | 2.13 | 4.25 | 2.21 | 34.19 | 33.81 | 34.25 | 3470 | 193-301 | 10.8 | 3 | 6 |
| 2266 | 19.12.2009 | 6:20 | 51.6121 | 0.0022 | 2.87 | 2.87 | 1.88 | 33.71 | 33.71 | 34.44 | 2774 | 0-71 | 7.1 | 1 | 6 |
| 2266 | 19.12.2009 | 6:10 | 51.6121 | 0.0022 | 1.32 | 2.87 | 1.88 | 33.88 | 33.71 | 34.44 | 2774 | 70-180 | 11 | 2 | 6 |
| 2266 | 19.12.2009 | 6:00 | 51.6121 | 0.0022 | 1.62 | 2.87 | 1.88 | 34.31 | 33.71 | 34.44 | 2774 | 181-300 | 11.9 | 3 | 6 |
| 2268 | 19.12.2009 | 15:20 | 52.2738 | -0.0035 | 2.91 | 2.9 | 1.94 | 33.73 | 33.74 | 34.47 | 2711 | 0-80 | 8 | 1 | 6 |
| 2268 | 19.12.2009 | 15:30 | 52.2738 | -0.0035 | 1.44 | 2.9 | 1.94 | 33.95 | 33.74 | 34.47 | 2711 | 80-170 | 9 | 2 | 6 |
| 2268 | 19.12.2009 | 15:10 | 52.2738 | -0.0035 | 1.34 | 2.9 | 1.94 | 34.4 | 33.74 | 34.47 | 2711 | 172-300 | 12.8 | 3 | 6 |
| 2270 | 19.12.2009 | 23:50 | 52.9303 | 0.1833 | 0.88 | 0.92 | 1.6 | 33.77 | 33.77 | 34.58 | 2070 | 0-90 | 9 | 1 | 6 |
| 2270 | 19.12.2009 | 23:40 | 52.9303 | 0.1833 | 0.24 | 0.92 | 1.6 | 34.06 | 33.77 | 34.58 | 2070 | 90-190 | 10 | 2 | 6 |
| 2270 | 19.12.2009 | 23:25 | 52.9303 | 0.1833 | 1.34 | 0.92 | 1.6 | 34.49 | 33.77 | 34.58 | 2070 | 193-300 | 11 | 3 | 6 |
| 2272 | 20.12.2009 | 9:15 | 53.5931 | 0.2667 | 1.25 | 1.29 | 1.61 | 33.74 | 33.75 | 34.58 | 2732 | 0-80 | 8 | 1 | 7 |
| 2272 | 20.12.2009 | 9:25 | 53.5931 | 0.2667 | 0.14 | 1.29 | 1.61 | 34.03 | 33.75 | 34.58 | 2732 | 80-201 | 12.1 | 2 | 7 |
| 2272 | 20.12.2009 | 9:05 | 53.5931 | 0.2667 | 1.34 | 1.29 | 1.61 | 34.49 | 33.75 | 34.58 | 2732 | 201-300 | 9.9 | 3 | 7 |
| 2273 | 20.12.2009 | 13:10 | 53.8925 | 0.0015 | 1.31 | 1.19 | 1.63 | 33.77 | 33.77 | 34.59 | 2437 | 0-95 | 9.5 | 1 | 7 |
| 2273 | 20.12.2009 | 13:20 | 53.8925 | 0.0015 | 0.31 | 1.19 | 1.63 | 34.04 | 33.77 | 34.59 | 2437 | 95-220 | 12.5 | 2 | 7 |
| 2273 | 20.12.2009 | 13:00 | 53.8925 | 0.0015 | 1.52 | 1.19 | 1.63 | 34.55 | 33.77 | 34.59 | 2437 | 227-310 | 8.3 | 3 | 7 |
| 2274 | 20.12.2009 | 17:50 | 54.2507 | -0.0027 | 1.01 | 1.09 | 1.63 | 33.77 | 33.77 | 34.59 | 2743 | 0-39 | 3.9 | 1 | 7 |
| 2274 | 20.12.2009 | 18:00 | 54.2507 | -0.0027 | 0.27 | 1.09 | 1.63 | 34.01 | 33.77 | 34.59 | 2743 | 40-203 | 16.3 | 2 | 7 |
| 2274 | 20.12.2009 | 17:40 | 54.2507 | -0.0027 | 1.39 | 1.09 | 1.63 | 34.51 | 33.77 | 34.59 | 2743 | 200-300 | 10 | 3 | 7 |
| 2275 | 20.12.2009 | 21:40 | 54.5837 | -0.0095 | 0.43 | 0.58 | 1.28 | 33.81 | 33.81 | 34.51 | 1273 | 0-80 | 8 | 1 | 7 |
| 2275 | 20.12.2009 | 21:30 | 54.5837 | -0.0095 | -0.81 | 0.58 | 1.28 | 33.94 | 33.81 | 34.51 | 1273 | 81-180 | 9.9 | 2 | 7 |
| 2275 | 20.12.2009 | 21:20 | 54.5837 | -0.0095 | 0.79 | 0.58 | 1.28 | 34.39 | 33.81 | 34.51 | 1273 | 183-300 | 11.7 | 3 | 7 |
| 2277 | 21.12.2009 | 5:20 | 55.2412 | 0.003 | 0.23 | 0.37 | 1.81 | 33.82 | 33.81 | 34.56 | 2780 | 0-80 | 8 | 1 | 7 |
| 2277 | 21.12.2009 | 5:10 | 55.2412 | 0.003 | -0.2 | 0.37 | 1.81 | 34.06 | 33.81 | 34.56 | 2780 | 80-180 | 10 | 2 | 7 |
| 2277 | 21.12.2009 | 4:55 | 55.2412 | 0.003 | 1.64 | 0.37 | 1.81 | 34.49 | 33.81 | 34.56 | 2780 | 185-310 | 12.5 | 3 | 7 |
| 2278 | 21.12.2009 | 10:30 | 55.5677 | 0.0022 | 0.2 | 0.3 | 1.5 | 33.88 | 33.89 | 34.55 | 3748 | 0-80 | 8 | 1 | 8 |
| 2278 | 21.12.2009 | 10:20 | 55.5677 | 0.0022 | -0.09 | 0.3 | 1.5 | 34.1 | 33.89 | 34.55 | 3748 | 80-180 | 10 | 2 | 8 |
| 2278 | 21.12.2009 | 10:10 | 55.5677 | 0.0022 | 1.48 | 0.3 | 1.5 | 34.48 | 33.89 | 34.55 | 3748 | 182-300 | 11.8 | 3 | 8 |
| 2279 | 21.12.2009 | 14:30 | 55.8997 | 0.0013 | 0.1 | 0.3 | 1.45 | 33.93 | 33.91 | 34.59 | 3671 | 0-90 | 9 | 1 | 8 |
| 2279 | 21.12.2009 | 14:20 | 55.8997 | 0.0013 | -0.39 | 0.3 | 1.45 | 34.13 | 33.91 | 34.59 | 3671 | 90-190 | 10 | 2 | 8 |
| 2279 | 21.12.2009 | 14:10 | 55.8997 | 0.0013 | 1.07 | 0.3 | 1.45 | 34.5 | 33.91 | 34.59 | 3671 | 191-300 | 10.9 | 3 | 8 |
| 2280 | 21.12.2009 | 21:10 | 56.2222 | 0.0023 | 0.16 | 0.29 | 0.5 | 34.09 | 34.04 | 34.57 | 3894 | 0-63 | 6.3 | 1 | 9 |
| 2280 | 21.12.2009 | 21:00 | 56.2222 | 0.0023 | -0.53 | 0.29 | 0.5 | 34.26 | 34.04 | 34.57 | 3894 | 63-205 | 14.2 | 2 | 9 |
| 2280 | 21.12.2009 | 20:50 | 56.2222 | 0.0023 | 0.09 | 0.29 | 0.5 | 34.5 | 34.04 | 34.57 | 3894 | 205-310 | 10.5 | 3 | 9 |
| 2281 | 22.12.2009 | 1:50 | 56.5618 | 0.0023 | 0.1 | 0.2 | 0.83 | 34.14 | 34.14 | 34.65 | 4200 | 0-70 | 7 | 1 | 9 |
| 2281 | 22.12.2009 | 1:30 | 56.5618 | 0.0023 | -0.28 | 0.2 | 0.83 | 34.34 | 34.14 | 34.65 | 4200 | 70-210 | 14 | 2 | 9 |
| 2281 | 22.12.2009 | 1:20 | 56.5618 | 0.0023 | 0.78 | 0.2 | 0.83 | 34.61 | 34.14 | 34.65 | 4200 | 210-300 | 9 | 3 | 9 |
| 2282 | 22.12.2009 | 8:20 | 56.8968 | -0.0098 | 0.01 | 0.02 | 0.59 | 34.19 | 34.18 | 34.64 | 3720 | 0-50 | 5 | 1 | 9 |
| 2282 | 22.12.2009 | 8:25 | 56.8968 | -0.0098 | -0.348 | 0.02 | 0.59 | 34.306 | 34.18 | 34.64 | 3720 | 50-200 | 15 | 2 | 9 |
| 2282 | 22.12.2009 | 8:05 | 56.8968 | -0.0098 | 0.47 | 0.02 | 0.59 | 34.6 | 34.18 | 34.64 | 3720 | 201-300 | 9.9 | 3 | 9 |
