# Supplementary material for: Biogeography of the Southern Ocean: environmental factors driving mesoplankton distribution South of Africa
Source: PeerJ. 2021 May 10;9:e11411. doi: 10.7717/peerj.11411 (PMC8117931; doi:10.7717/peerj.11411)
Supplement: Supplemental Information 2 [file peerj-09-11411-s002.docx]

Appendix 2. Full list of taxa ordered according to their percent contribution to the total mesoplankton abundance

| **Taxa** | **Contribution, %** |
| --- | --- |
| 1. *Oithona similis* Claus, 1866 | 49.20 |
| 1. Calanoida copepodites indet. | 11.86 |
| 1. Euphausiidae nauplii | 4.91 |
| 1. *Ctenocalanus citer* Heron & Bowman, 1971 | 4.89 |
| 1. Foraminifera | 4.62 |
| 1. Ova (copepoda) | 3.06 |
| 1. *Microcalanus pusillus* Sars G.O., 1903 | 2.82 |
| 1. Appendicularia | 2.70 |
| 1. Radiolaria | 2.04 |
| 1. *Clausocalanus brevipes* Frost & Fleminger, 1968 | 1.36 |
| 1. *Triconia antarctica* ♂ (Heron, 1977) | 1.29 |
| 1. *Clausocalanus pergens* Farran, 1926 | 1.10 |
| 1. *Paracalanus parvus parvus* (Claus, 1863) | 0.81 |
| 1. *Oncaea* sp. | 0.67 |
| 1. *Pleuromamma gracilis* Claus, 1863 | 0.65 |
| 1. Ostracoda | 0.56 |
| 1. *Metridia lucens lucens*Boeck, 1865 | 0.49 |
| 1. Chaetognatha | 0.45 |
| 1. *Acartia longiremis* (Lilljeborg, 1853) | 0.43 |
| 1. Copepoda nauplii | 0.43 |
| 1. *Calanus simillimus* Giesbrecht, 1902 | 0.42 |
| 1. *Calocalanus contractus* Farran, 1926 | 0.39 |
| 1. Pteropoda | 0.39 |
| 1. *Scolecithricella minor* (Brady, 1883) | 0.35 |
| 1. *Oithona plumifera* Baird, 1843 | 0.34 |
| 1. *Ctenocalanus vanus* Giesbrecht, 1888 | 0.31 |
| 1. *Calocalanus styliremis* Giesbrecht, 1888 | 0.30 |
| 1. *Calanus propinquus* Brady, 1883 | 0.29 |
| 1. *Euchaeta marina* (Prestandrea, 1833) | 0.25 |
| 1. *Triconia antarctica* ♀ (Heron, 1977) | 0.23 |
| 1. *Onychocorycaeus giesbrechti* (Dahl F., 1894) | 0.19 |
| 1. Euphausiacea calyptopis | 0.19 |
| 1. Salpidae | 0.18 |
| 1. *Clausocalanus laticeps* Farran, 1929 | 0.15 |
| 1. Euphausiacea furcilia | 0.14 |
| 1. *Rhincalanus gigas* Brady, 1883 | 0.11 |
| 1. *Nannocalanus minor* (Claus, 1863) | 0.09 |
| 1. Siphonophora | 0.09 |
| 1. Polychaeta | 0.08 |
| 1. *Calanoides acutus* (Giesbrecht, 1902) | 0.08 |
| 1. *Microsetella rosea* (Dana, 1847) | 0.07 |
| 1. *Pareucalanus langae* (Fleminger, 1973) | 0.07 |
| 1. *Calocalanus tenuis* Farran, 1926 | 0.06 |
| 1. *Thysanoessa longipes* Brandt, 1851 | 0.06 |
| 1. *Pleuromamma xiphias* (Giesbrecht, 1889) | 0.06 |
| 1. *Heterorhabdus spinifrons* (Claus, 1863) | 0.06 |
| 1. *Hyperia* sp. | 0.05 |
| 1. *Scaphocalanus brevicornis* (Sars G.O., 1900) | 0.05 |
| 1. *Spinocalanus magnus* Wolfenden, 1904 | 0.03 |
| 1. *Mesocalanus tenuicornis* (Dana, 1849) | 0.03 |
| 1. *Metridia gerlachei* Giesbrecht, 1902 | 0.03 |
| 1. *Lucicutia flavicornis* (Claus, 1863) | 0.02 |
| 1. *Heterorhabdus papilliger* (Claus, 1863) | 0.02 |
| 1. *Euchaeta acuta* Giesbrecht, 1893 | 0.01 |
| 1. *Calocalanus* sp. | 0.01 |
| 1. *Lucicutia lucida* Farran, 1908 | 0.01 |
| 1. *Centropages bradyi* Wheeler, 1900 | 0.01 |
| 1. *Pleuromamma quadrungulata* (Dahl F., 1893) | 0.01 |
| 1. *Haloptilus oxycephalus* (Giesbrecht, 1889) | 0.01 |
| 1. *Euchirella rostrata* (Claus, 1866) | 0.01 |
| 1. *Pareuchaeta pseudotonsa* (Fontaine, 1967) | 0.01 |
| 1. *Phronima* sp. | 0.01 |
| 1. *Aetideus armatus* (Boeck, 1872) | 0.01 |
| 1. Gastropoda larvae | 0.01 |
| 1. Medusae | 0.01 |
| 1. *Tomopteris* sp. | 0.01 |
| 1. Amphipoda larvae | <0.01 |
| 1. Amphipoda indet. | <0.01 |
| 1. Fish larvae | <0.01 |
| 1. *Candacia maxima* Vervoort, 1957 | <0.01 |
| 1. *Aetideus acutus* Farran, 1929 | <0.01 |
| 1. *Scolecithricella minor* (Brady, 1883) | <0.01 |
| 1. *Haloptilus longicornis* (Claus, 1863) | <0.01 |
| 1. *Oculosetella gracilis* (Dana, 1849) | <0.01 |
| 1. *Neomormonilla minor* (Giesbrecht, 1891) | <0.01 |
| 1. *Sapphirina angusta* Dana, 1849 | <0.01 |
| 1. *Rhincalanus nasutus* Giesbrecht, 1888 | <0.01 |
| 1. *Clausocalanus furcatus* (Brady, 1883) | <0.01 |
| 1. Isopoda | <0.01 |
| 1. *Paraheterorhabdus robustus* (Farran, 1908) | <0.01 |
| 1. *Themisto* sp. | <0.01 |
| 1. *Gaetanus tenuispinus* (Sars G.O., 1900) | <0.01 |
| 1. *Scaphocalanus* sp. | <0.01 |
| 1. *Scolecithrix danae* (Lubbock, 1856) | <0.01 |
| 1. *Urocorycaeus furcifer* (Claus, 1863) | <0.01 |
| 1. *Sapphirina* sp. | <0.01 |
| 1. *Oikopleura* sp. | <0.01 |
| 1. *Vibilia* sp. | <0.01 |
| 1. *Scolecithrix bradyi* Giesbrecht, 1888 | <0.01 |
| 1. *Gaetanus minor* Farran, 1905 | <0.01 |
| 1. *Scina* sp. | <0.01 |
| 1. *Goniopsyllus rostratus* Brady, 1883 | <0.01 |
| 1. Euphausiacea indet. | <0.01 |
| 1. *Aetideus giesbrechti* Cleve, 1904 | <0.01 |
| 1. Cirripedia nauplii | <0.01 |
| 1. *Oithona nana* Giesbrecht, 1893 | <0.01 |
| 1. *Corycaeus speciosus* Dana, 1849 | <0.01 |
| 1. Decapoda larvae | <0.01 |
| 1. *Candacia cheirura* Cleve, 1904 | <0.01 |
| 1. *Aetideus australis* (Vervoort, 1957) | <0.01 |
| 1. Bivalvia larvae | <0.01 |
| 1. *Pareuchaeta biloba* Farran, 1929 | <0.01 |
| 1. Polychaeta larvae | <0.01 |
| 1. Bryozoa larvae | <0.01 |
| 1. *Lucicutia curta* Farran, 1905 | <0.01 |
| 1. *Calocalanus pavo* (Dana, 1852) | <0.01 |
| 1. *Lophothrix latipes* (Scott T., 1894) | <0.01 |
| 1. *Candacia catula* (Giesbrecht, 1889) | <0.01 |
| 1. *Pyrosoma* | <0.01 |
| 1. *Aegisthus mucronatus* Giesbrecht, 1891 | <0.01 |
| 1. *Onychocorycaeus giesbrechti* (Dahl F., 1894) | <0.01 |
| 1. *Lucicutia ovalis* (Giesbrecht, 1889) | <0.01 |
| 1. *Haloptilus ocellatus* Wolfenden, 1905 | <0.01 |
| 1. Echinodermata larvae | <0.01 |
| 1. *Eucalanus elongatus elongatus* (Dana, 1848) | <0.01 |
| 1. *Macrosetella gracilis* (Dana, 1847) | <0.01 |
| 1. *Pareuchaeta gracilis* (Sars G.O., 1905) | <0.01 |
| 1. *Pareuchaeta* sp. | <0.01 |
| 1. *Lucicutia clausi* (Giesbrecht, 1889) | <0.01 |
| 1. *Scottocalanus securifrons* (Scott T., 1894) | <0.01 |
| 1. *Phaenna spinifera* Claus, 1863 | <0.01 |
| 1. *Pareuchaeta sarsi* (Farran, 1908) | <0.01 |
| 1. *Centropages gracilis* (Dana, 1849) | <0.01 |
| 1. *Farranula rostrata* (Claus, 1863) | <0.01 |
| 1. *Pareuchaeta bisinuata* (Sars G.O., 1907) | <0.01 |
| 1. *Candacia* sp. | <0.01 |
| 1. *Undeuchaeta plumosa* (Lubbock, 1856) | <0.01 |
| 1. Cephalopoda | <0.01 |
| 1. *Neocalanus gracilis* (Dana, 1852) | <0.01 |
| 1. *Euchirella truncata* Esterly, 1911 | <0.01 |
| 1. *Pleuromamma robusta* (Dahl F., 1893) | <0.01 |
| 1. *Agetus limbatus* (Brady, 1883) | <0.01 |
| 1. *Scottocalanus persecans* (Giesbrecht, 1895) | <0.01 |
| 1. *Undinula vulgaris* (Dana, 1849) | <0.01 |
| 1. *Clausocalanus arcuicornis* (Dana, 1849) | <0.01 |
| 1. *Paraheterorhabdus compactus* (Sars G.O., 1900) | <0.01 |
| 1. *Lubbockia squillimana* Claus, 1863 | <0.01 |
| 1. *Eupronoe* sp. | <0.01 |
| 1. *Centropages violaceus* (Claus, 1863) | <0.01 |
| 1. *Chirundina streetsii* Giesbrecht, 1895 | <0.01 |
| 1. *Subeucalanus monachus* (Giesbrecht, 1888) | <0.01 |
| 1. *Pareuchaeta tonsa* (Giesbrecht, 1895) | <0.01 |
| 1. *Cyphocaris* sp. | <0.01 |
| 1. *Aetideopsis rostrata* Sars G.O., 1903 | <0.01 |
| 1. *Lubbockia aculeata* Giesbrecht, 1891 | <0.01 |
| 1. *Echinomysis* sp. | <0.01 |
| 1. *Paraheterorhabdus robustus* (Farran, 1908) | <0.01 |
| 1. *Aetideopsis carinata* Bradford, 1969 | <0.01 |
| 1. *Heterostylites major* (Dahl F., 1894) | <0.01 |
| 1. *Euchirella truncata* Esterly, 1911 | <0.01 |
| 1. *Candacia varicans* (Giesbrecht, 1893) | <0.01 |
| 1. *Subeucalanus longiceps* (Matthews, 1925) | <0.01 |
| 1. *Chiridius gracilis* Farran, 1908 | <0.01 |
| 1. *Haloptilus spiniceps* (Giesbrecht, 1893) | <0.01 |
| 1. *Rhabdosoma* sp. | <0.01 |
| 1. *Streetsia* sp. | <0.01 |
| 1. *Eucalanus hyalinus* (Claus, 1866) | <0.01 |
| 1. *Euchirella* sp. | <0.01 |
| 1. *Gaetanus robustus* Sars G.O., 1905 | <0.01 |
| 1. *Spinocalanus brevicaudatus* Brodsky, 1950 | <0.01 |
| 1. *Gaetanus miles* Giesbrecht, 1888 | <0.01 |
| 1. *Nullosetigera helgae* (Farran, 1908) | <0.01 |
| 1. *Valdiviella minor* Wolfenden, 1911 | <0.01 |
