# Supplementary material for: Biogeography of the Southern Ocean: environmental factors driving mesoplankton distribution South of Africa
Source: PeerJ. 2021 May 10;9:e11411. doi: 10.7717/peerj.11411 (PMC8117931; doi:10.7717/peerj.11411)

Appendix 3. Results of the one-way ANOSIM analyses with zones bounded by four hydrological fronts as a grouping factor for the upper mixed layer (A), intermediate layer (B), deep layer (C), and whole 0-300 m layer. Hydrological front coding (vertical black lines): Subtropical Front (STF); Subantarctic Front (SAF-M), Polar Front (PF), Southern Boundary (SB). Solid and dotted lines indicate statistically significant and insignificant boundaries, respectively.


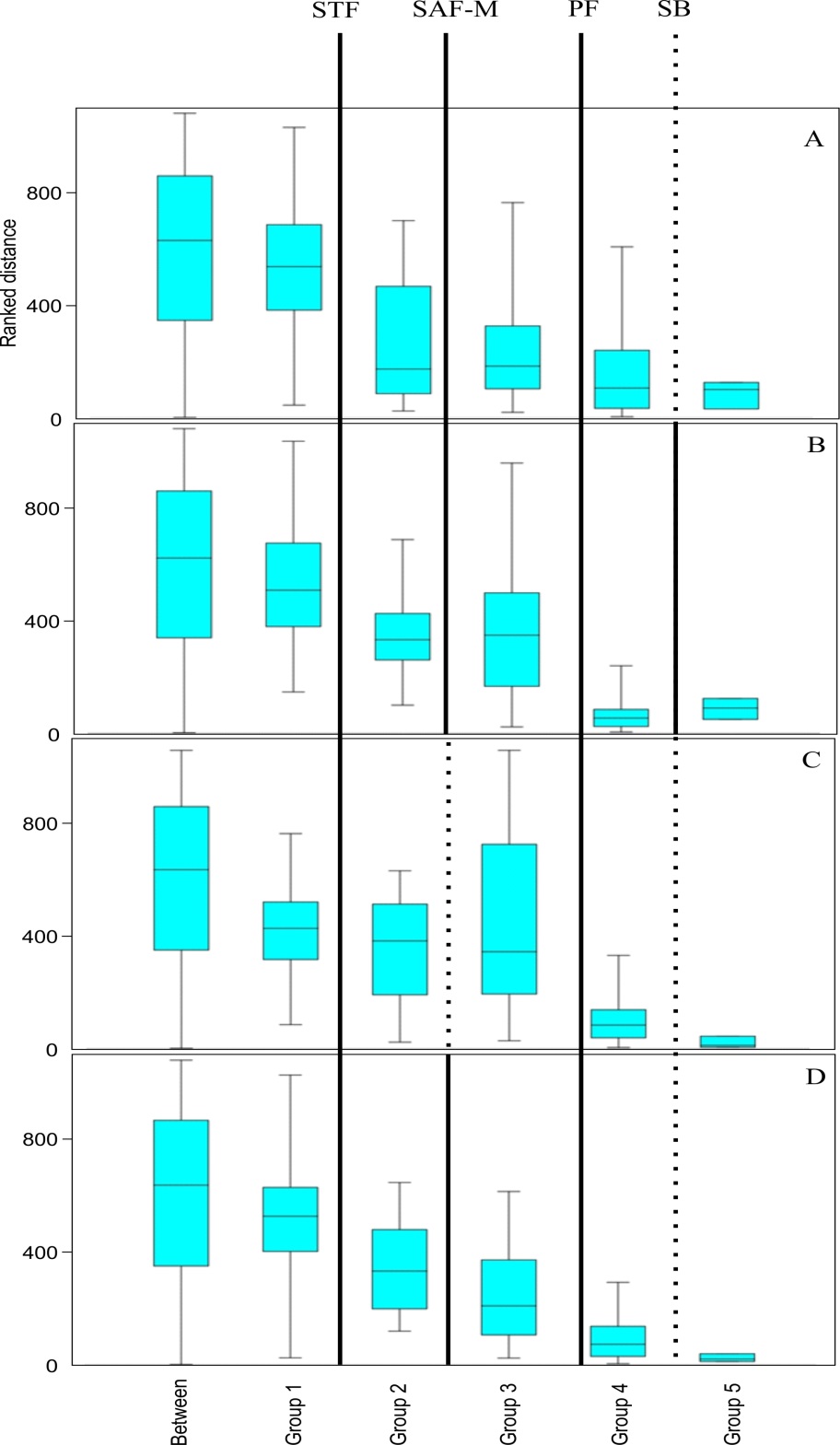

Supplement: Supplemental Information 3 — Hydrological front coding (vertical black lines): Subtropical Front (STF); Subantarctic Front (SAF-M), Polar Front (PF), Southern Boundary (SB). Solid and dotted lines indicate statistically significant and insignificant boundaries, respectively. [file peerj-09-11411-s003.docx]
