# Supplementary material for: Biogeography of the Southern Ocean: environmental factors driving mesoplankton distribution South of Africa
Source: PeerJ. 2021 May 10;9:e11411. doi: 10.7717/peerj.11411 (PMC8117931; doi:10.7717/peerj.11411)
Supplement: Supplemental Information 4 — Tested layers: upper mixed (Layer 1), intermediate (Layer 2), deep (Layer 3), and whole 0-300 m layer (Layer 0). Zones: north of STF (1), between STF and SAF-M (2), between SAF-M and PF (3), between PF and SB (4), and south of SB (5). Statistically significant boundaries between neighburing zones are in bold. [file peerj-09-11411-s004.docx]

Appendix 4. Results of ANOSIM tests used to indicate the difference between plankton assemblages bounded by hydrological fronts (Bray-Curtis quantitative index used). Tested layers: upper mixed (Layer 1), intermediate (Layer 2), deep (Layer 3), and whole 0-300 m layer (Layer 0). Zones: north of STF (1), between STF and SAF-M (2), between SAF-M and PF (3), between PF and SB (4), and south of SB (5). Statistically significant boundaries between neighburing zones are in bold.

| **Layer 1** | | | | |
| --- | --- | --- | --- | --- |
|  | 2 | 3 | 4 | 5 |
| 1 | **0.0426** | **0.0017** | **0.0003** | **0.4101** |
| 2 |  | 0.5418 | 0.1864 | **0.0325** |
| 3 |  |  | 0.1389 | 0.1111 |
| 4 |  |  |  | **0.0486** |
| **Layer 2** | | | | |
|  | 2 | 3 | 4 | 5 |
| 1 | **0.009** | **0.0027** | **0.0001** | 0.0676 |
| 2 |  | 0.5524 | **0.0001** | **0.0363** |
| 3 |  |  | **0.0003** | 0.4505 |
| 4 |  |  |  | 0.5181 |
| **Layer 3** | | | | |
|  | 2 | 3 | 4 | 5 |
| 1 | **0.0013** | **0.0001** | **0.0001** | **0.0022** |
| 2 |  | 0.6545 | **0.0002** | **0.0251** |
| 3 |  |  | **0.0006** | 0.4835 |
| 4 |  |  |  | 0.2414 |
