# Supplementary material for: Biogeography of the Southern Ocean: environmental factors driving mesoplankton distribution South of Africa
Source: PeerJ. 2021 May 10;9:e11411. doi: 10.7717/peerj.11411 (PMC8117931; doi:10.7717/peerj.11411)

Appendix 5. Results of the one-way ANOSIM analyses with zones bounded by eight dynamic jets as a grouping factor for the upper mixed layer (A), intermediate layer (B), deep layer (C), and the whole 0-300 m layer. Hydrological jet coding (vertical black lines): Subtropical Front (STF); Subantarctic Front (SAF), three branches (northern SAF-N, middle SAF-M and southern SAF-S); Polar Front (PF), merged branches; Southern Front (SACCF), two branches (northern SACCF-N and southern SACCF-S); Southern Boundary (SB). Solid and dotted lines indicate statistically significant and insignificant boundaries, respectively.

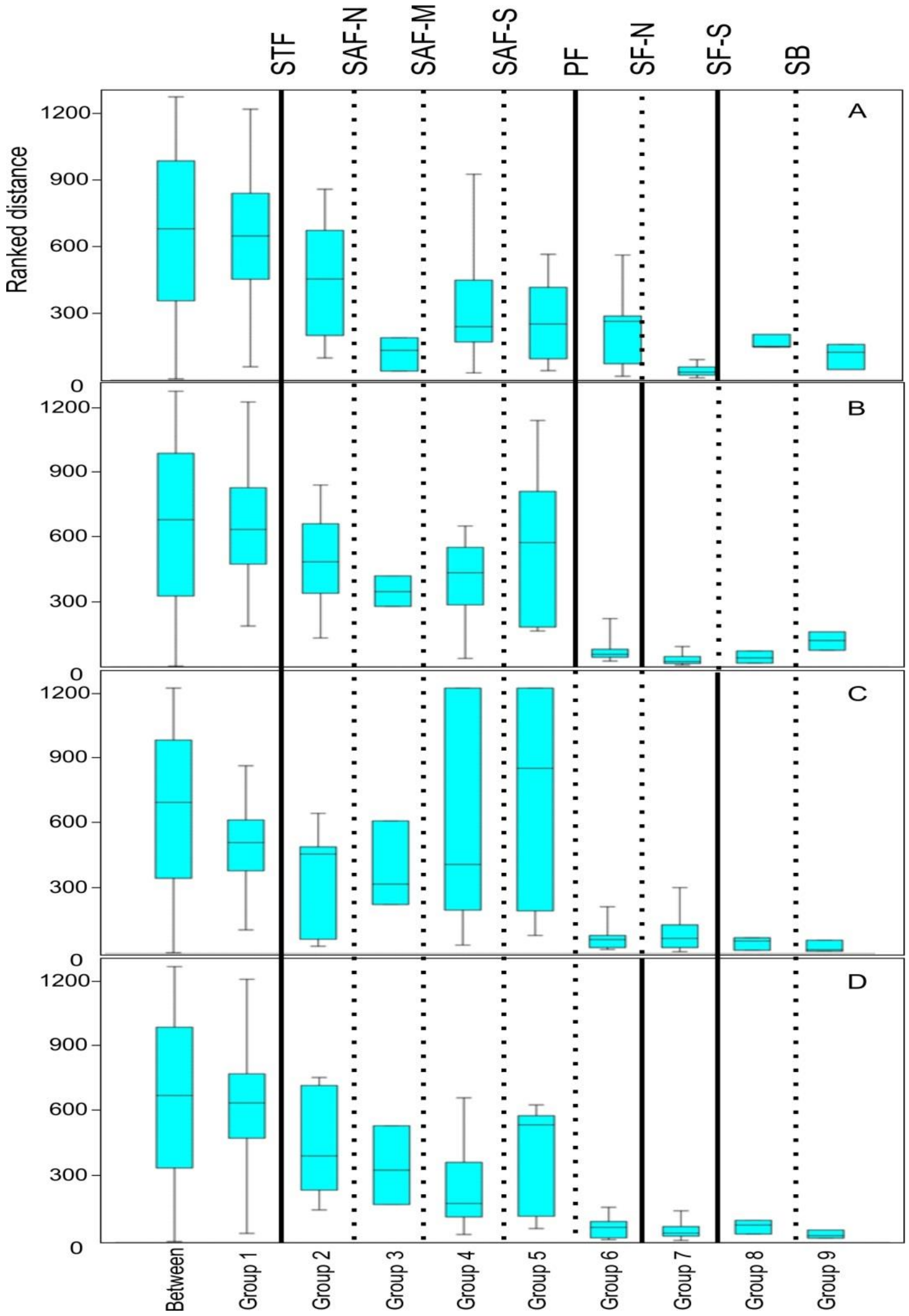

Supplement: Supplemental Information 5 — Hydrological jet coding (vertical black lines): Subtropical Front (STF); Subantarctic Front (SAF), three branches (northern SAF-N, middle SAF-M and southern SAF-S); Polar Front (PF), merged branches; Southern Front (SACCF), two branches (northern SACCF-N and southern SACCF-S); Southern Boundary (SB). Solid and dotted lines indicate statistically significant and insignificant boundaries, respectively. [file peerj-09-11411-s005.pdf]
