# Supplementary material for: Biogeography of the Southern Ocean: environmental factors driving mesoplankton distribution South of Africa
Source: PeerJ. 2021 May 10;9:e11411. doi: 10.7717/peerj.11411 (PMC8117931; doi:10.7717/peerj.11411)
Supplement: Supplemental Information 6 — Tested layers: upper mixed (Layer 1), intermediate (Layer 2), deep (Layer 3), and the entire 0-300 m layer (Layer 0). Zones: north of STF (1), between STF and SAF-N (2), between SAF-N and SAF-M (3), between SAF-M and SAF-S (4), between SAF-S and PF (5), between PF and SACCF-N (6), between SACCF-N and SACCF-S (7), between SACCF-S and (8), and south of SB (9). Statistically significant boundaries between neighboring zones are in bold. [file peerj-09-11411-s006.docx]

Appendix 6. Results of the ANOSIM tests determining the difference between plankton asseblages bounded by dynamic jets (Bray-Curtis quantitative index). Tested layers: upper mixed (Layer 1), intermediate (Layer 2), deep (Layer 3), and the entire 0-300 m layer (Layer 0). Zones: north of STF (1), between STF and SAF-N (2), between SAF-N and SAF-M (3), between SAF-M and SAF-S (4), between SAF-S and PF (5), between PF and SACCF-N (6), between SACCF-N and SACCF-S (7), between SACCF-S and (8), and south of SB (9). Statistically significant boundaries between neighboring zones are in bold.

| **Layer 1** | | | | | | | | |
| --- | --- | --- | --- | --- | --- | --- | --- | --- |
|  | 2 | 3 | 4 | 5 | 6 | 7 | 8 | 9 |
| 1 | 0.1646 | 0.184 | **0.044** | 0.1301 | **0.0205** | **0.0136** | 0.4193 | 0.4147 |
| 2 |  | 0.4956 | 0.3146 | 0.4005 | 0.0902 | **0.02** | 0.3242 | 0.0554 |
| 3 |  |  | 0.744 | 0.3856 | 0.16 | **0.0226** | 0.099 | 0.1001 |
| 4 |  |  |  | 0.2596 | 0.0892 | **0.0311** | **0.5767** | **0.1705** |
| 5 |  |  |  |  | 0.4901 | 0.1157 | 0.3725 | 0.3994 |
| 6 |  |  |  |  |  | 0.424 | 0.0564 | 0.0899 |
| 7 |  |  |  |  |  |  | **0.0228** | **0.0119** |
| 8 |  |  |  |  |  |  |  | 0.801 |
| **Layer 2** | | | | | | | | |
|  | 2 | 3 | 4 | 5 | 6 | 7 | 8 | 9 |
| 1 | 0.1347 | 0.0691 | **0.003** | **0.0073** | **0.002** | **0.001** | **0.0408** | 0.061 |
| 2 |  | 0.5997 | 0.2306 | 0.1106 | **0.0162** | **0.0017** | 0.1043 | 0.0862 |
| 3 |  |  | 0.7291 | 0.7525 | 0.0505 | **0.0117** | 0.1013 | 0.1049 |
| 4 |  |  |  | 0.2027 | **0.0271** | **0.0136** | 0.0962 | 0.0778 |
| 5 |  |  |  |  | 0.0972 | **0.027** | 0.3535 | 0.4666 |
| 6 |  |  |  |  |  | 0.4195 | 0.4285 | 0.2649 |
| 7 |  |  |  |  |  |  | 0.502 | 0.1907 |
| 8 |  |  |  |  |  |  |  | 0.7033 |
| **Layer 3** | | | | | | | | |
|  | 2 | 3 | 4 | 5 | 6 | 7 | 8 | 9 |
| 1 | **0.0162** | **0.002** | **0.0003** | **0.0008** | **0.0014** | **0.0001** | **0.0038** | **0.0023** |
| 2 |  | 0.3813 | 0.5469 | 0.2026 | **0.0227** | **0.0056** | 0.0568 | 0.0546 |
| 3 |  |  | 0.7782 | 0.4729 | **0.0349** | **0.0139** | 0.0978 | 0.1045 |
| 4 |  |  |  | 0.2659 | 0.3969 | 0.0551 | 0.4015 | 0.5066 |
| 5 |  |  |  |  | 0.0649 | **0.0046** | 0.2026 | 0.2339 |
| 6 |  |  |  |  |  | 0.257 | 0.0881 | 0.0537 |
| 7 |  |  |  |  |  |  | 0.4616 | 0.1353 |
| 8 |  |  |  |  |  |  |  | 1 |
