# Supplementary material for: Biogeography of the Southern Ocean: environmental factors driving mesoplankton distribution South of Africa
Source: PeerJ. 2021 May 10;9:e11411. doi: 10.7717/peerj.11411 (PMC8117931; doi:10.7717/peerj.11411)
Supplement: Supplemental Information 7 — All clades and nodes are robust (p <0.05), and red lines indicate minimal similarity between two marked clades (exact value unrecognizable). Colored figures on bottom indicate robust clusters. [file peerj-09-11411-s007.docx]

Appendix. 7. Results from the cluster analysis (Bray-Curtis qualitative index) for samples collected within the upper mixed layer. All clades and nodes are robust (p <0.05), and red lines indicate minimal similarity between two marked clades (exact value unrecognizable). Colored figures on bottom indicate robust clusters.


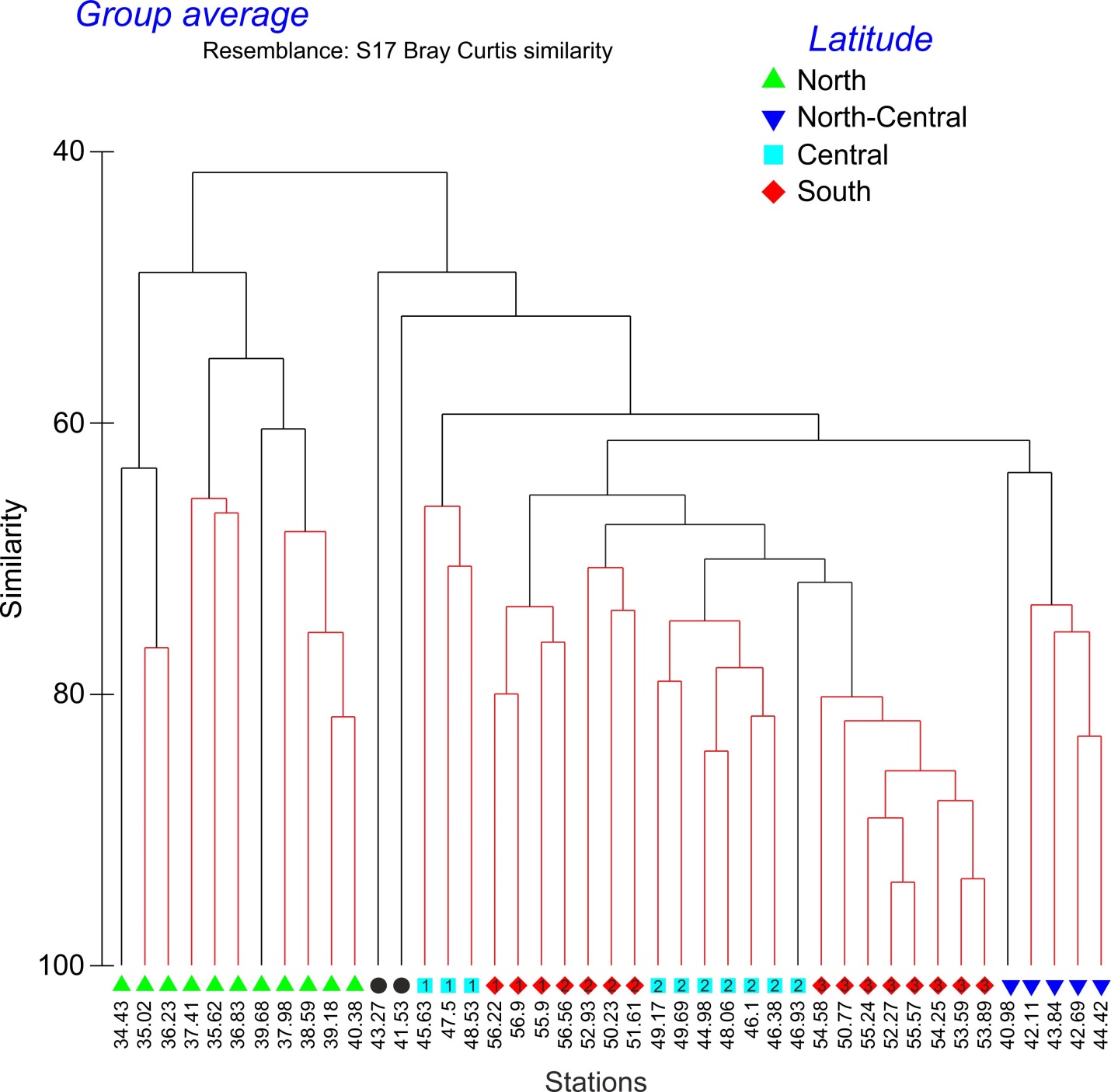


Latitudes, ºS
