# Supplementary material for: Biogeography of the Southern Ocean: environmental factors driving mesoplankton distribution South of Africa
Source: PeerJ. 2021 May 10;9:e11411. doi: 10.7717/peerj.11411 (PMC8117931; doi:10.7717/peerj.11411)
Supplement: Supplemental Information 9 — Colored figures on bottom indicate robust clusters. [file peerj-09-11411-s009.pdf]

Appendix 9. Results from the cluster analysis (Bray-Curtis qualitative index) for samples collected within the deep layer. Colored figures on bottom indicate robust clusters.

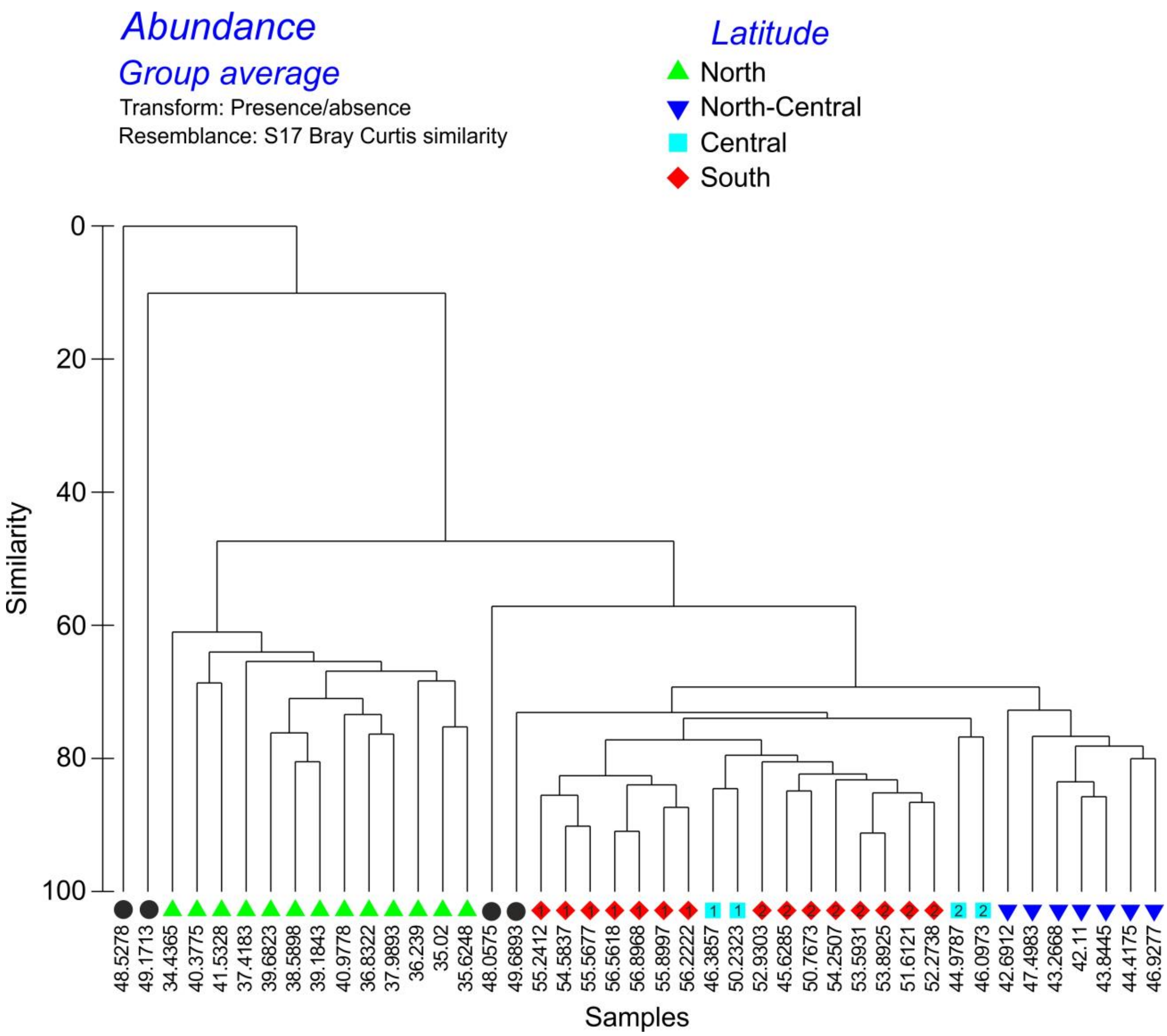

Latitudes, ºS
