# Supplementary material for: Biogeography of the Southern Ocean: environmental factors driving mesoplankton distribution South of Africa
Source: PeerJ. 2021 May 10;9:e11411. doi: 10.7717/peerj.11411 (PMC8117931; doi:10.7717/peerj.11411)
Supplement: Supplemental Information 10 — Colors indicate groups diverging at certain similarity levels. [file peerj-09-11411-s010.docx]

Appendix 10. Results from the cluster analysis (Bray-Curtis quantitative similarity index) from samples collected within the upper mixed layer (Layer 1) and intermediate layer (Layer 2). Colors indicate groups diverging at certain similarity levels.


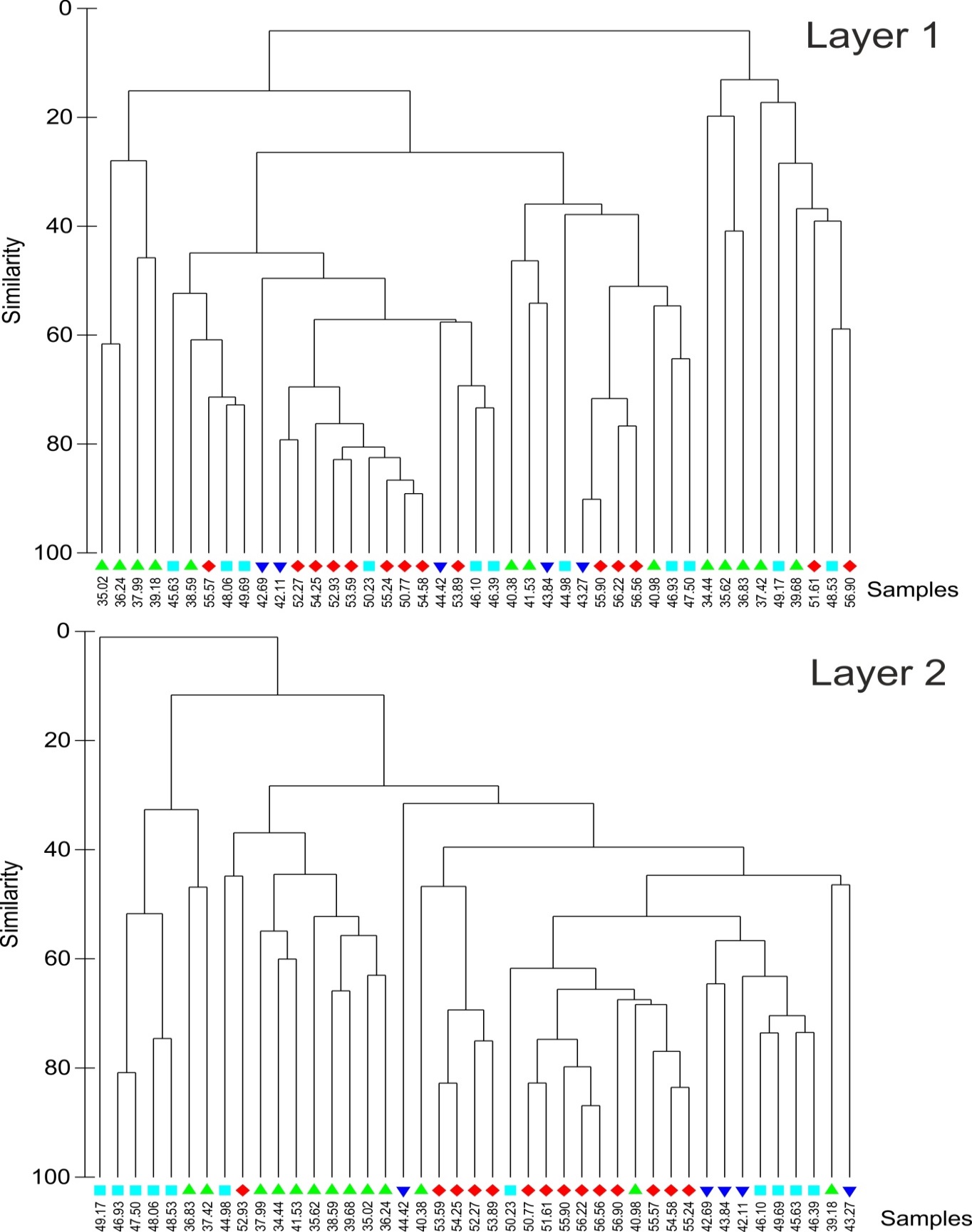


Latitudes, ºS
